# Clinical evaluation of General Electric new Swiftscan solution in bone scintigraphy on NaI-camera: A head to head comparison with Siemens Symbia

**DOI:** 10.1371/journal.pone.0222490

**Published:** 2019-09-19

**Authors:** F. Thibault, M. Bailly, G. Le Rouzic, G. Metrard

**Affiliations:** Nuclear Medicine Department, CHR Orléans, ORLEANS, FRANCE; Cleveland Clinic, UNITED STATES

## Abstract

**Purpose:**

The General Electric (GE) Swiftscan solution combines a new Low Energy High Resolution and Sensitivity collimator (LEHRS) with image processing (Clarity 2D) and tomographic step and shoot continuous mode. The aim of this study was to compare clinical and physical performances of this new technology in bone scintigraphy.

**Methods:**

Physical phantom measurements were performed using GE LEHRS, GE Low Energy High Resolution (LEHR) and Siemens LEHR collimators. These measurements were associated with a prospective clinical study. Sixty-seven patients referred for bone scintigraphy were enrolled from February to July 2018. Each patient underwent two acquisitions consecutively on GE and Siemens gamma camera, using respectively Swiftscan solution and LEHR collimator.

**Results:**

On planar acquisitions, maximum sensitivity was 100 cts/MBq for Siemens LEHR. GE SwiftScan LEHRS and GE LEHR maximum sensitivity were respectively 9% and 22% lower. Using Clarity 2D, GE Swiftscan LEHRS spatial resolution was the best with 9.2 mm versus 10.1 mm and 10.6 mm for GE LEHR and Siemens LEHR collimators. In tomographic mode, the sensitivity of GE Swiftscan solution was superior to both LEHR systems (16% and 25% respectively for Siemens and GE). There was no significant difference in spatial resolution. In clinical use, signal was higher on Siemens system and noise was lower on GE Swiftscan solution. Contrast-to-noise ratios were not significantly different between the two systems. There was a significant image quality improvement with GE SwiftScan in planar images and in whole body scan. No significant difference in image quality was observed on SPECT images.

**Conclusion:**

New GE SwiftScan collimator design improved sensitivity compared to “classical” GE LEHR collimator without compromising resolution. GE SwiftScan solution enhances planar image quality with a better Clarity 2D resolution recovery and noise treatment. In SPECT mode, GE SwiftScan solution improves volumetric sensitivity without significant impact on image quality, and could lead to time or dose reduction.

## Introduction

Most scintigraphic exams use Tc-99m radiolabeled pharmaceuticals and require Low-Energy High Resolution (LEHR) collimators. Collimator design is always a compromise between spatial resolution and sensitivity **[1]**. Considering continuous informatic progresses, a greater part of image quality is due to post-processing.

General Electric Healthcare (GE Healthcare, Milwaukee, WI, USA) recently commercialized a SwiftScan® solution for planar and SPECT acquisitions with a pair of new Low Energy High Resolution and Sensitivity collimators (LEHRS).

SwiftScan solution combines LEHRS collimators and Clarity 2D image enhancement algorithm for resolution recovery and noise reduction in planar acquisitions. Clarity 2D is a 3 steps post-processing workflow on 2D images, automatically applied just after acquisition. This workflow is composed of a noise reduction with adaptive edge preservation, contrast enhancement and filtered image blending with native image. In SPECT mode, LEHRS collimators with step-and-shoot continuous mode enhance sensitivity, allowing data acquisitions during heads rotation. Clarity 2D isn’t available in SPECT mode.

Only few studies compared the performances of gamma camera collimators in clinical use because it is easier and more reproductible to perform this kind of study on phantoms **[2, 3]**.

The aim of this prospective study was to compare physical and clinical performances of SwiftScan solution in bone scintigraphy.

## Materials and methods

This study has been approved by French National Ethics Committee SUD-MEDITERRANEE 1 (internal number 1824) and registered under 2018-A00622-53. This study has also been declared to Clinical Trial (n° NCT03497078).

### Phantom study

All physical measurements were performed on Siemens Symbia T2 with LEHR collimators and on Discovery NM670 (GE Healthcare), with both “classical” LEHR and new SwiftScan pre-production LEHRS collimators **[4]**. Energy window acquisition was 140 ± 7.5%.

Time acquisition was adapted in order to have the same number of phantom emission counts in each experiment.

In planar mode, sensitivity and spatial resolution were measured according to National Electrical Manufacturers Association (NEMA) guidelines NU1-2000 **[5]**. Spatial resolution was evaluated by measuring full width at half maximum (FWHM) of the linear spread function (LSF) with ImageJ software (National Institute of mental Health, NIH, Bethesda, MD, USA). In order to evaluate depth dependence, LSF measurements were realized in air and through PolyMethylMethAcrylate (PMMA) attenuating medium.

In tomographic mode, volumetric sensitivity and spatial resolution were measured according to NEMA NU1-2000 **[5]**. Image quality was evaluated according to NEMA NU2-2012 **[6]** guideline (contrast of 8). Projections were acquired according to routine protocols used in the Nuclear Medicine Department and in order to have the same number of phantom emission counts in each experiment. Volumes were reconstructed using a manufacturer-independent software (OASIS, Segamicorp, Columbia, MD, USA), considering spatial resolution, depth dependence and Chang attenuation correction.

### Patients

In this prospective monocentric study, sixty-seven patients referred for bone scintigraphy (32 men, 35 women; mean age 56 ± 15.5 years) were enrolled from February to July 2018.

Exclusion criteria were patients under 18 years old, painful patients, pregnancy, breastfeeding, kidney failure and recent bisphosphonate treatment.

As mentioned before, this study was approved by an Ethics Committees and was performed according to the principles of the 1964 Declaration of Helsinki and its later amendments. Written informed consent was obtained from all patients.

Data were collected in a specific studybook.

To avoid disturbing the clinical routine activity in our department and to avoid any loss of chance in the event of the study process being stopped (pain …), the order in which patients passed on each camera respected the random assignment of appointments, without specific randomization. The first acquisition was always performed on the scheduled camera.

**Fig 1:**
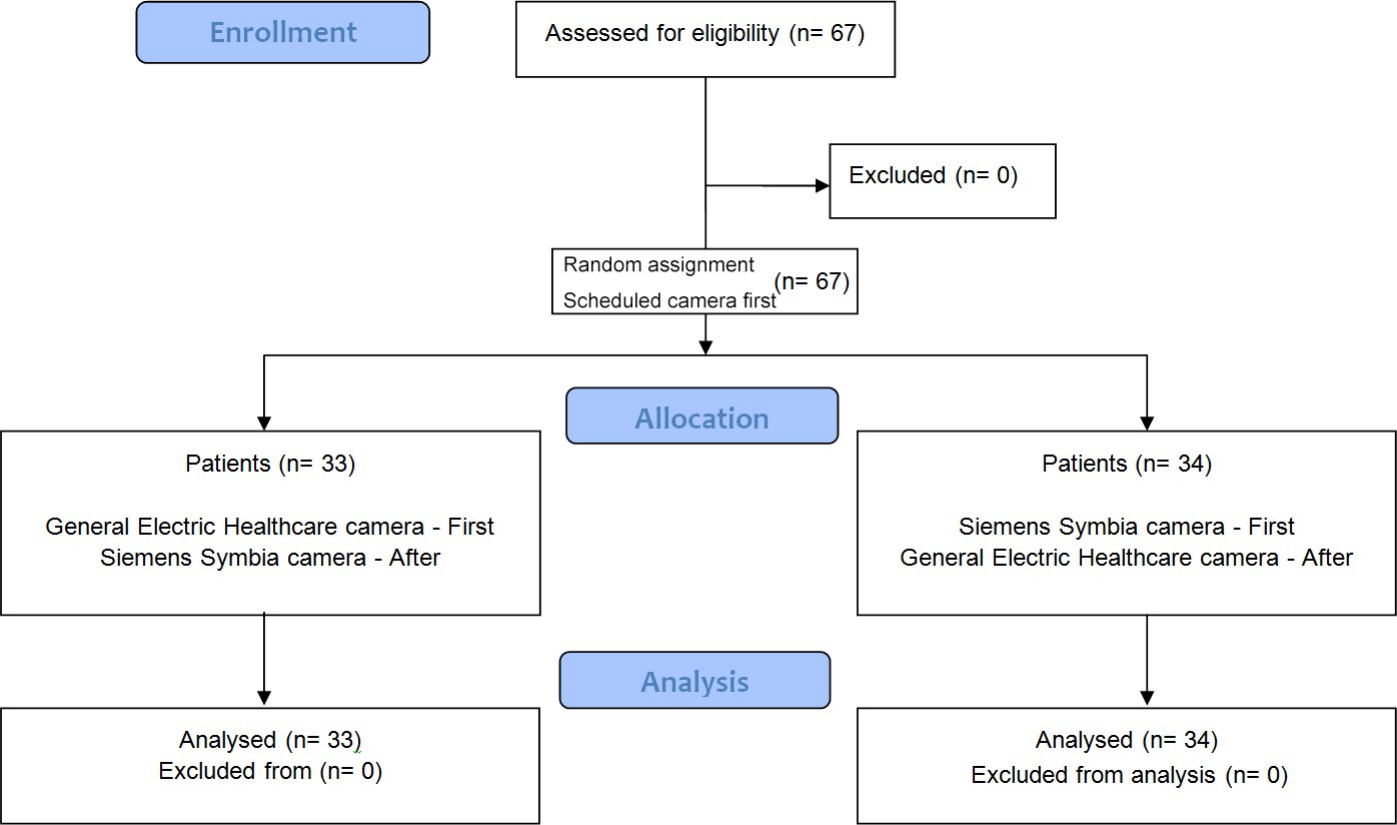
Study flowchart.

Patient demographics are listed in **Table 1**. Study Flowchart is described on **Fig 1**.

**Table 1:** Patient demographics.

Number of patients	67
Gender (Male : Female)	32 : 35
Mean age ± SD (y)	56 ± 15.5 (18–84)
BMI (kg/m^2^)	26.8 ± 4.9 (19.27–43.21)
Indication for bone scintigraphy:	
Rheumatology	28
Oncology	21
Orthopaedics	18
Number of SPECT acquisitions (% of total exams)	36 (53.7)
First acquisition:	
GE SwiftScan first n (%)	33 (49.3)
Siemens Symbia first n (%)	34 (50.7)

### Patients imaging protocol

All patients underwent whole body scan (WBS) and at least one planar static acquisition 2 hours after an intravenous injection of 9.25 MBq/kg Tc-99m-methylene diphosphonate (MDP). Each WBS and planar acquisitions were performed on both dual-head GE Discovery NM670 with SwiftScan solution and dual-head Siemens LEHR Symbia T2 system. The order of passage for both cameras was aleatory with camera availability.

Acquisition protocol was at least a 18 cm.min^-1^ WBS and a 3 min planar acquisition with 256x256 matrix. GE SwiftScan acquisitions were realized using recommended manufacturer parameters with a 40% weighted-image Clarity 2D.

For clinical purposes, an additional SPECT/CT was realized in 36 patients with low-dose CT for attenuation correction and anatomical co-registration. SPECT angular sampling was 6° with 20s time per projection, zoom factor of 1.23 and 128x128 matrix. Only one CT was performed in order to reduce patient dosimetry.

SPECT images were reconstructed using a three-dimensional (3D) iterative ordered subsets expectation maximization algorithm (OSEM) with recommended manufacturer parameters and resolution recovery for each camera (OSEM 3D 4 iterations 6 subsets, no post-filter for GE Swiftscan acquisitions; OSEM 3D (FLASH 3D) 4 iterations 8 subsets, 5mm FWHM Gaussian post-filter, pixel size of 4.8x4.8 mm for Siemens Symbia), and with independent Oasis software (4 iterations 10 subsets for GE SwiftScan acquisitions; 5 iterations 8 subsets for Siemens Symbia—Butterworth post-filter 6/0.4 for both). Despite a different number of projections (60 and 64 respectively for GE and Siemens systems), those two reconstructions were comparable because of a constant iterations-subsets product. No attenuation or scatter corrections were performed.

For each image (WBS, planar and SPECT), acquisition time on the second system was adapted to compensate the radioactive decay between scans (mean time shift of 46.7 ± 20.8 min).

### Image evaluation

Images were anonymised by a medical resident.

Blinded-image analysis was performed by two board-certified Nuclear Medicine physicians from the Nuclear Medicine Department.

Image quality was evaluated using a 5-point Likert scale as described in **Table 2**. Image quality could be a subjective preference, that is why this scale was defined with objective criteria: visualization of interosseous spaces, bone to soft tissues contrast that referred to semi-quantitative resolution and contrast. Observers scored WBS, additional planar view and SPECT separately.

The percentage of exams judged to be of good quality (scores 4 and 5) was compared for each collimator and for each type of image.

**Table 2:** 5-point Likert scale with description.

Score	
**5**	**Diagnostic quality image**: excellent image quality and resolution (e.g. excellent skeletal uptake, background noise almost non-existent, excellent visualization of bone surfaces and interosseous spaces)
**4**	**Diagnostic quality image**: good image quality and resolution (e.g. good skeletal uptake, low background noise, good visualization of bone surfaces and interosseous spaces)
**3**	**Acceptable image quality**: acceptable image quality and resolution (e.g. acceptable skeletal uptake, moderate background noise, vague visualization of bone surfaces and interosseous spaces)
**2**	**Non-optimal quality**: limited clinical information (e.g. low skeletal uptake, important background noise, poor visualization of bone surfaces and interosseous spaces)
**1**	**Non-diagnostic quality**: low or no bone uptake, important background noise).

For inter-observer agreement, a score of at least 4 was set as diagnostic image quality for statistical analysis.

Moreover, quantitative measurements were performed on geometric average of WBS acquisitions for both systems. Regions of interest (ROIs) were drawn on femoral diaphysis and sacroiliac joints, and mirror ROIs were duplicated over adjacent soft tissues. Mean pixel counts and standard deviation were measured. Absolute contrast, relative contrast and contrast-to-noise ratios (CNR) were calculated. Absolute contrast was defined as the absolute difference between bone and soft tissue ROI mean values and relative contrast as absolute contrast to soft tissue mean value. CNR was calculated as absolute contrast to square root of the sum of squared standard deviation values.

$$CNR=\frac{Absolute\;contrast}{\sqrt{\sigma_{1}{}^{2}+\sigma_{2}{}^{2}}}$$

Where σ_1_ and σ_2_ are respectively standard deviation of bone and soft tissue ROIs.

### Statistical analysis

For statistical analysis, data were collected on Excel spread sheet and analyzed using the Statistical Package for Social Sciences (SPSS v25; IBM corporation, Armonk, NY, USA).

Sample size was calculated with expected performances of the new system (R software v 1.1.423, RStudio Inc, Boston, MA, USA). A non-inferiority margin had been set at 12% with a rate of quality images of 97% with the reference camera, a level of significance of 5% and a power of 80%.

For hypothesis testing, a non-parametric Wilcoxon signed rank test was used for ordinal and non-Gaussian distribution variables (variable’s distribution was tested with a Kolmogorov-Smirnov normality test).

Inter-observer agreement was assessed with a Cohen’s kappa test. The level of agreement was classified into six Landis and Koch categories **[7]**.

Level of significance was set to 5%.

## Results

### Phantom experiments

#### Planar mode

**Fig 2:**
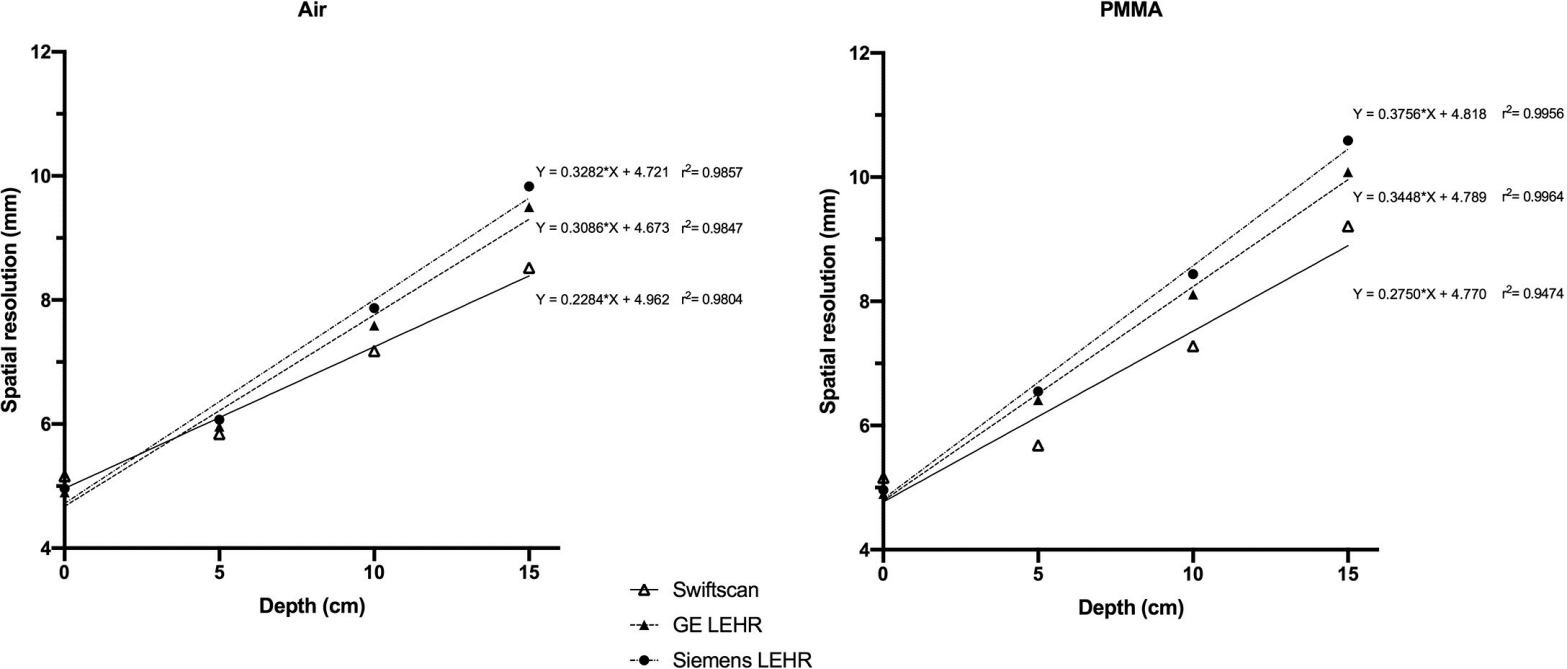
Spatial resolution in air and PMMA. In contact with detector, resolution of GE Swiftscan is lower than GE and Siemens LEHR systems. With clarity 2D, resolution recovery is better so that GE Swiftscan system resolution is better from 5 cm distance. The difference is more important in PMMA which is closer to clinical attenuation.

Maximum sensitivity was 100 cts/MBq for Siemens LEHR. GE SwiftScan LEHRS and GE LEHR were respectively 9% and 22% lower. FWHM of the linear spread function (LSF), at source contact, was equivalent for both LEHR collimators and 5% higher for GE SwiftScan LEHRS collimators. Nevertheless, thanks to Swiftscan Clarity 2D, FWHM degradation of LSF was less important when the distance with collimator increased. With 15 cm of diffusing material, spatial resolution was 9.2 mm for GE SwiftScan LEHRS, 10.1 mm for GE LEHR and 10.6 mm for Siemens LEHR collimators (**Fig 2**).

#### Tomographic mode

**Fig 3:**
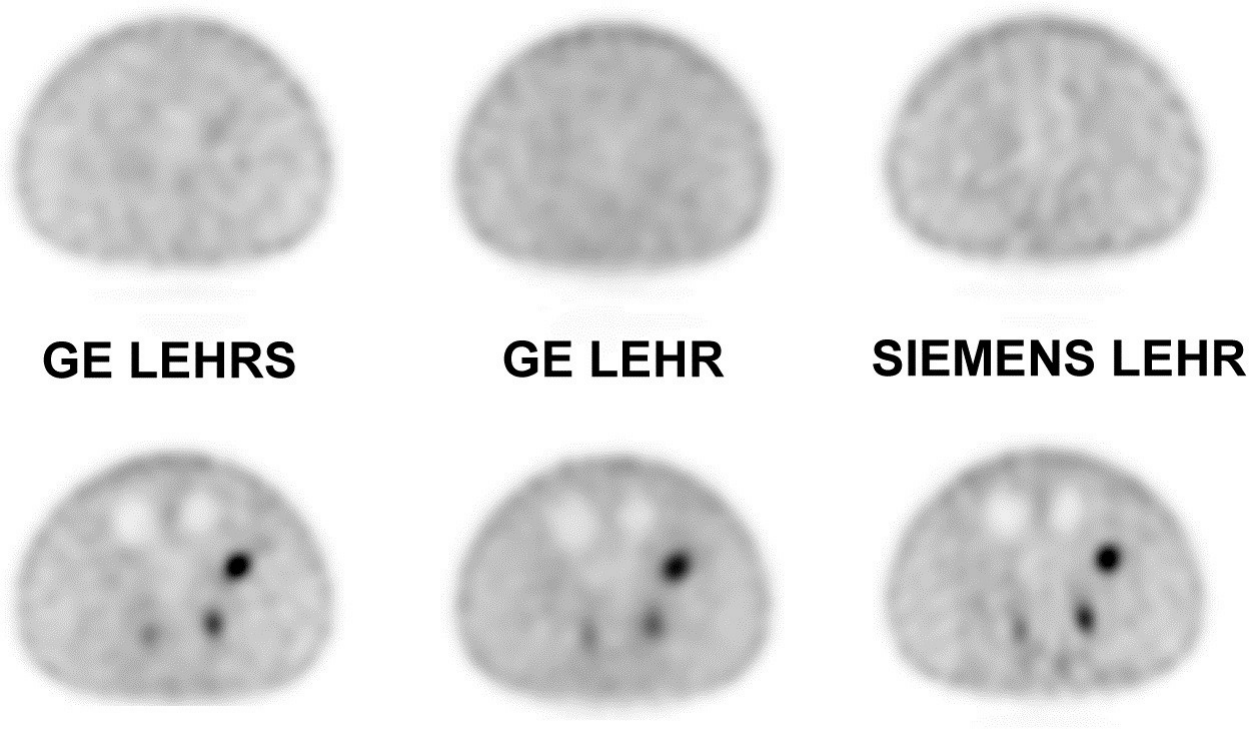
Phantom data. The best signal intensity is observed on Siemens LEHR acquisitions but noise is better controlled with GE Swiftscan solution.

GE SwiftScan volumetric sensitivity was superior to both LEHR systems (16% and 25% respectively for Siemens and GE). Spatial resolution was equivalent for the 3 collimators. The biggest hot sphere contrast recovery was equivalent for both GE collimators (41% for LEHR and 39.3% for SwiftScan LEHRS) and superior to Siemens LEHR (35.7%). GE SwiftScan solution provided a better background variability (7.9%) than both LEHR systems (10.1% and 9.8% respectively for GE and Siemens) (**Fig 3**).

### Clinical results

Statistical analysis on WBS ROIs showed a significantly better bone signal for Siemens with a mean ROI counts of 274.8 ± 157.1 on femurs and 1764.0 ± 719.4 on sacroiliac joints, versus respectively 183.1 ± 102.4 and 1238.0 ± 486.8 for GE SwiftScan LEHRS images (p<0.001).

Using GE Swiftscan solution, we measured less noise in thigh and hip soft tissues with a standard deviation of respectively 46.7 ± 20.7 and 250.6 ± 88.6 for GE SwiftScan and 98.0 ± 41.5 and 472.3 ± 175.3 for Siemens WBS images (p<0.001).

Absolute bone contrasts were significantly better for Siemens images with femoral ratios of 219.4 ± 122.7 and sacroiliac ratios of 1694.7 ± 704.7, versus respectively 143.2 ± 78.8 and 1180.3 ± 480.9 for GE SwiftScan images (p<0.001).

Relative bone contrasts were also higher for Siemens images, with femoral ratios of 5.7 ± 3.7 and sacroiliac ratios of 40.7 ± 39.8, versus respectively 4.8 ± 2.8 and 32.7 ± 30.5 for GE SwiftScan (p<0.05).

**Fig 4:**
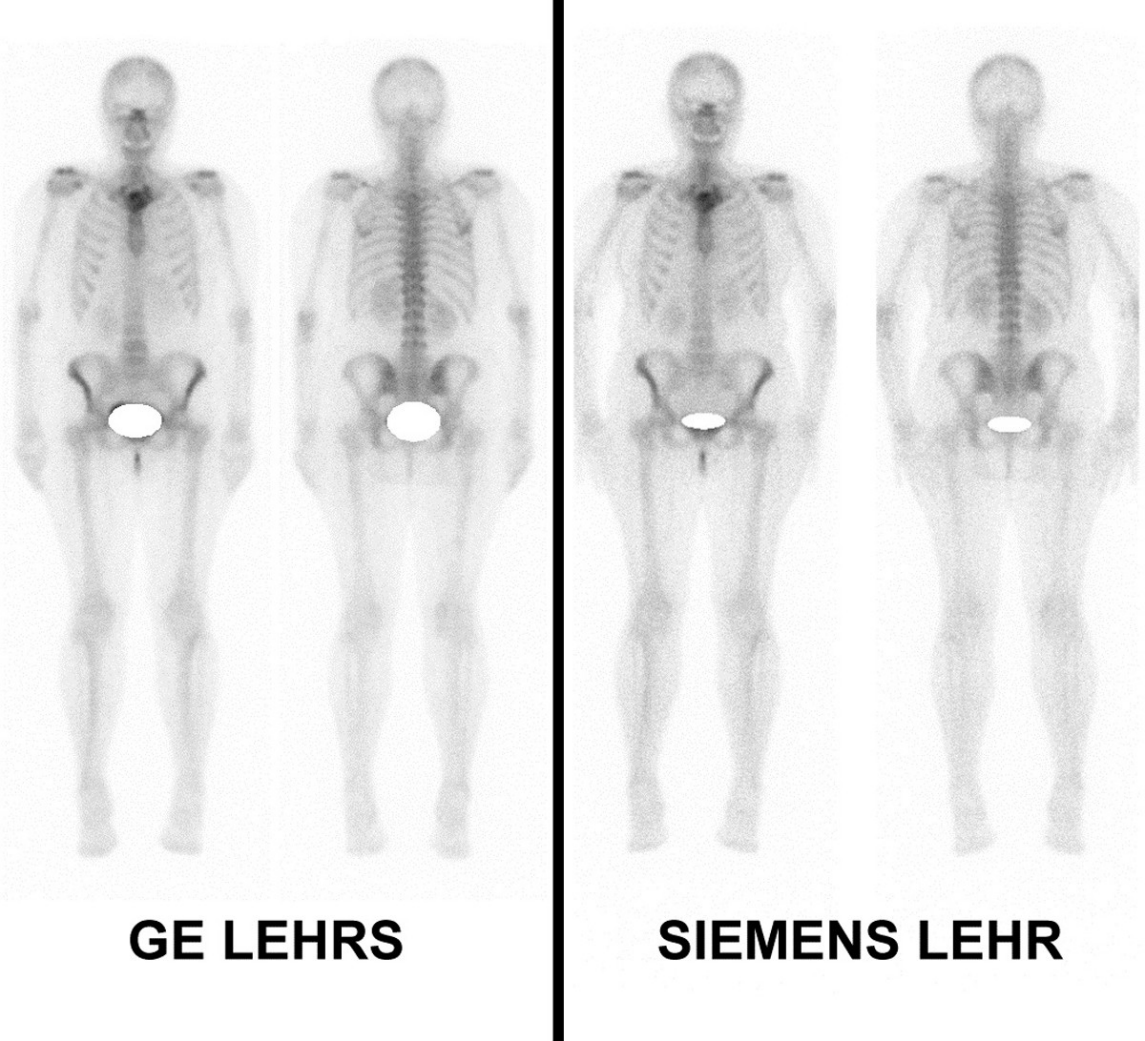
Whole body scan acquisitions in bone scintigraphy. Visually, heterogeneity in soft tissues is more important on Siemens LEHR images with more granulations in images. On quantification, with a 400 pixels region of interest on left axillary soft tissues, the standard deviation was measured at 1.79 for GE Swiftscan LEHRS and 2.72 for Siemens Symbia LEHR. We also observe a better delineation of each lumbar vertebra and left sternoclavicular joint with GE Swiftscan LEHRS.

**Fig 5:**
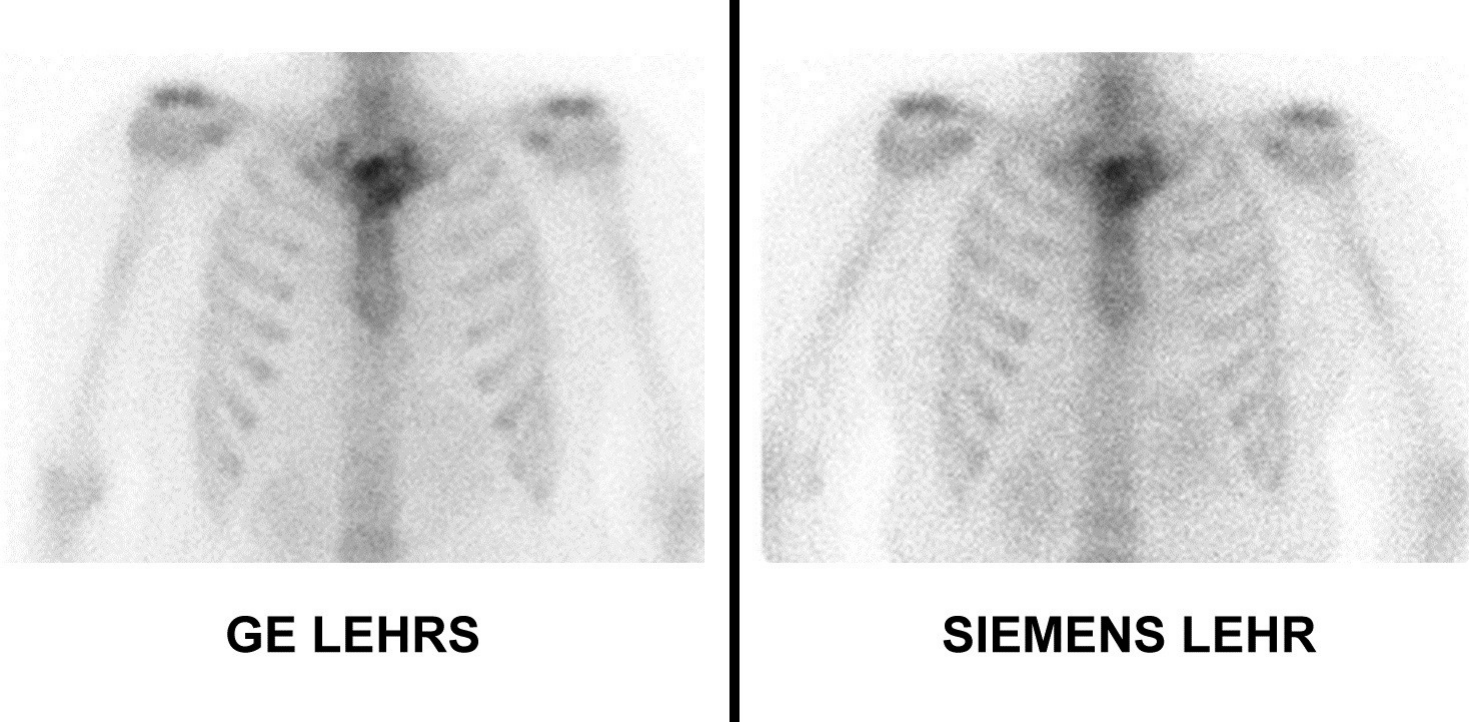
Planar acquisitions in bone scintigraphy.

There was no significant difference between GE SwiftScan and Siemens images for contrast-to-noise ratios: respectively 0.77 ± 0.10 and 0.79 ± 0.11 for femoral CNR (p = 0.10), and 0.95 ± 0.04 and 0.95 ± 0.06 for sacroiliac CNR (p = 0.33) (**Figs 4 and 5**).

Results are listed in **Table 3**.

**Table 3:** Signal, noise and contrast results.

	Siemens Symbia T2	Swiftscan	p value	Siemens Symbia T2	Swiftscan	p value
	Mean	Mean		SD	SD	
Femur bone signal	274.8	183.1	p<0.001	157.1	102.4	p<0.001
Sacroiliac bone signal	1764.0	1238.0	p<0.001	719.4	486.8	p<0.001
Absolute femoral bone contrast	219.4	143.2	p<0.001	122.7	78.8	
Absolute sacroiliac bone contrast	1694.7	1180.3	p<0.001	704.7	480.9	
Relative femoral bone contrast	5.7	4.8	p<0.05	3.7	2.8	
Relative sacroiliac bone contrast	40.7	32.7	p<0.05	39.8	30.5	
Femoral contrast to noise ratios	0.77	0.79	p = 0.10	0.10	0.11	
Sacroiliac contrast to noise ratios	0.95	0.95	p = 0.33	0.04	0.06	

We also find less heterogeneity in GE Swiftscan soft tissues planar images. On quantification, with a 400 pixels region of interest on left axillary soft tissues, the standard deviation was measured at 2.01 for GE Swiftscan LEHRS and 2.94 for Siemens Symbia LEHR. Delineation of lumbar vertebrae and left sternoclavicular joint is also better with GE Swiftscan LEHRS.

**Fig 6:**
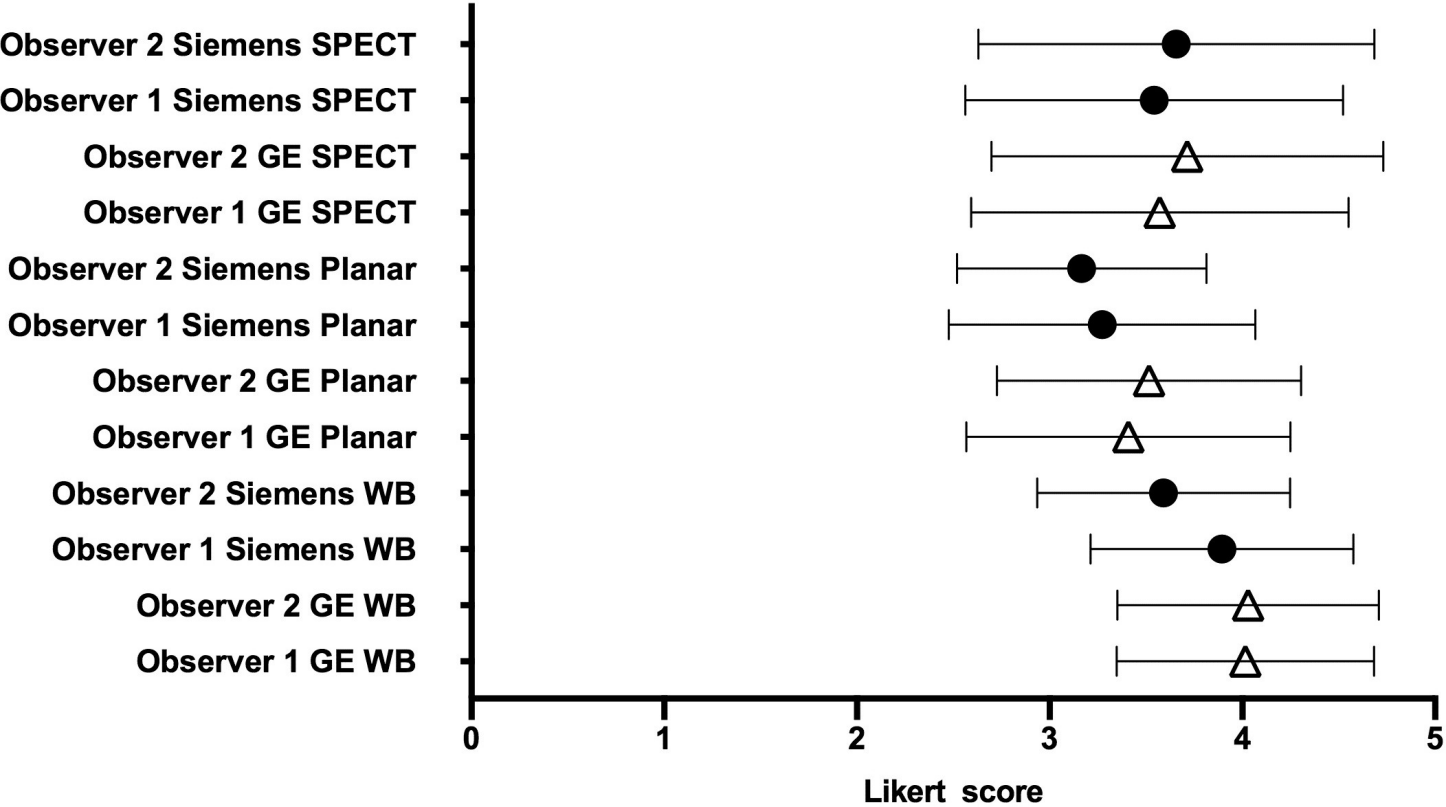
Bland-Altman representation of image scoring.

Results of image scoring for WBS, planar images and SPECT acquisitions for both systems are shown in **Fig 6**.

**Fig 7:**
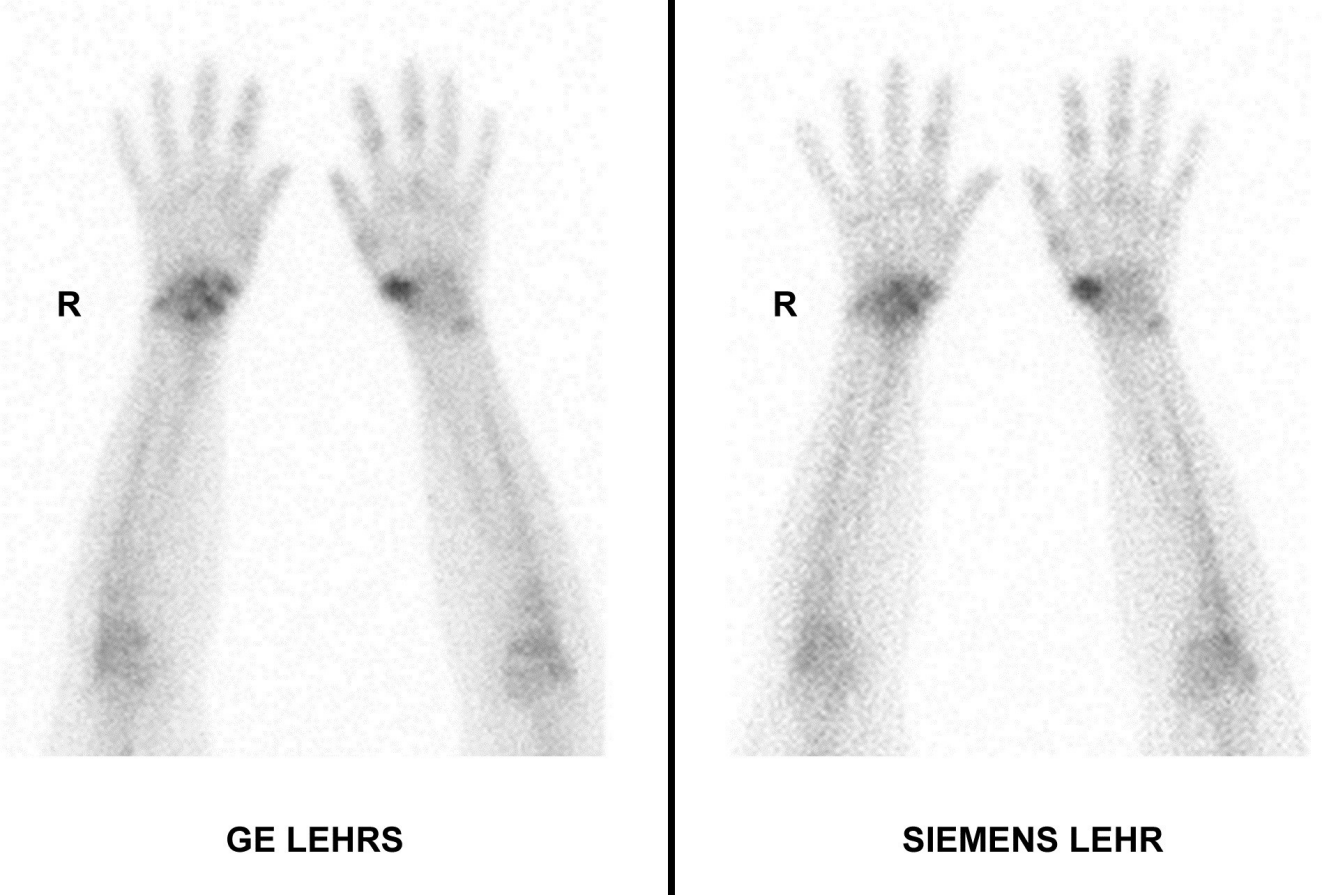
Hands planar acquisition in contact with collimator. More details are observed in the right carp on GE Swiftscan image.

There was a significant image quality improvement with GE SwiftScan solution on planar images for both observers (p<0.001), and on WBS only for observer 2 (p<0.05 versus p = 0.20 for observer 1) (**Fig 7)**.

**Fig 8:**
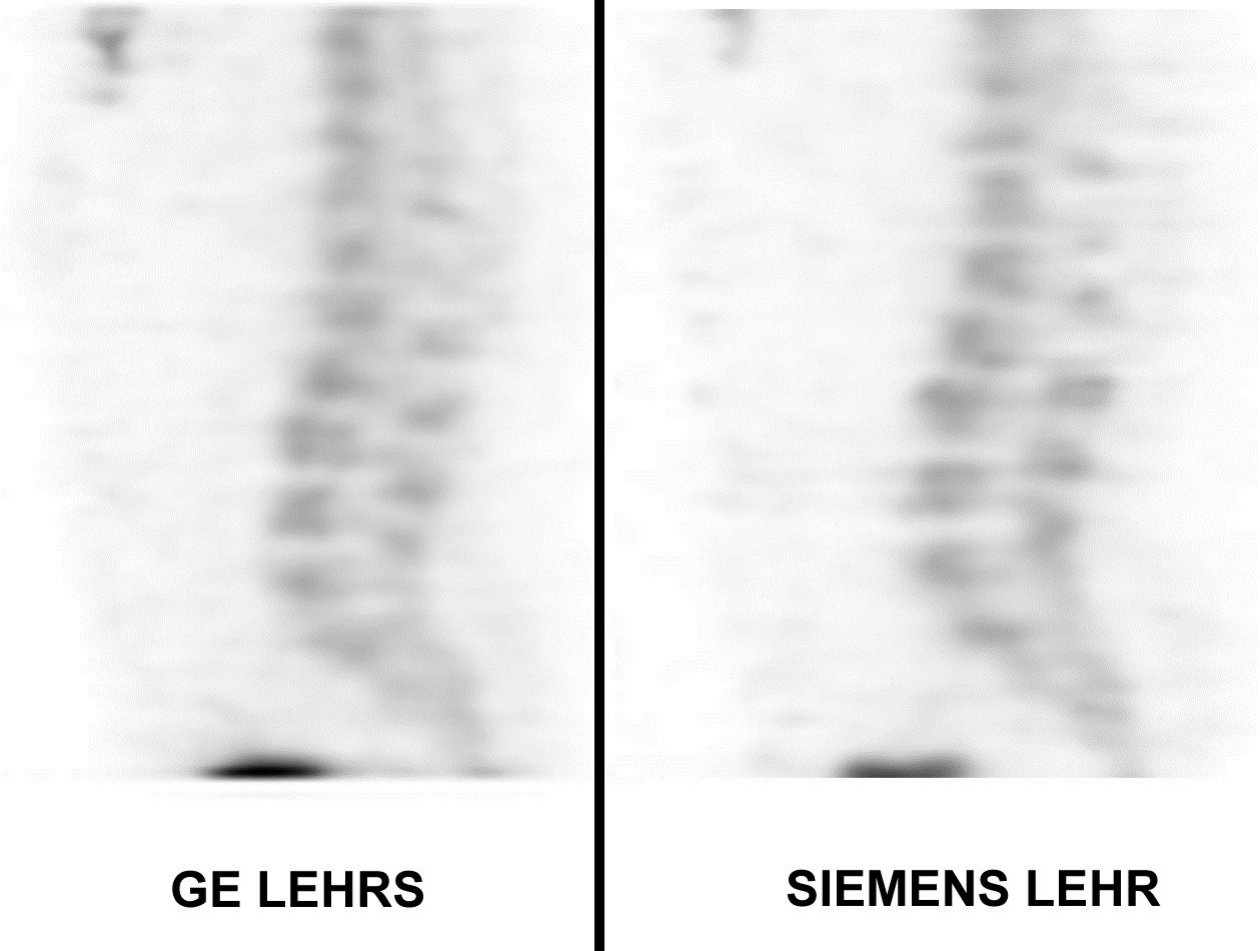
Lumbar SPECT. No difference in image quality was noticed between the two systems.

No statistical difference in image quality was observed on SPECT images between the two systems, independently of the reconstruction method and software (**Fig 8**).

Inter-observer agreement showed a substantial agreement for planar (κ = 0.728 for GE SwiftScan and 0.636 for Siemens) and SPECT images with Oasis reconstructions (κ = 0.722 for GE SwiftScan and 0.775 for Siemens). There was an almost perfect inter-observer agreement for SPECT images reconstructed with manufacturer workstations (κ = 0.827 for GE SwiftScan and 0.889 for Siemens). For WBS, inter-observer agreement was substantial for GE SwiftScan images (κ = 0.783) and moderate for Siemens images (κ = 0.489).

We didn’t find any significant difference in image quality between patients who were first scanned on GE system and those on Siemens system.

Results are listed in **Table 4**.

**Table 4:** Inter-observer agreement.

**SWIFTSCAN PLANAR**		**OBSERVER 2**			
		**Score ≤ 3**	**Score ≥ 4**	**TOTAL**	
**OBSERVER 1**	**Score ≤ 3**	33	5	38	k = 0.728
	**Score ≥ 4**	4	25	29	
**TOTAL**		37	30	67	
**SIEMENS PLANAR**		**OBSERVER 2**			
		**Score ≤ 3**	**Score ≥ 4**	**TOTAL**	
**OBSERVER 1**	**Score ≤ 3**	43	2	45	k = 0.636
	**Score ≥ 4**	8	14	22	
**TOTAL**		51	16	67	
**SWIFTSCAN WHOLEBODY**		**OBSERVER 2**			
		**Score ≤ 3**	**Score ≥ 4**	**TOTAL**	
**OBSERVER 1**	**Score ≤ 3**	9	3	12	k = 0.783
	**Score ≥ 4**	1	54	55	
**TOTAL**		10	57	67	
**SIEMENS WHOLEBODY**		**OBSERVER 2**			
		**Score ≤ 3**	**Score ≥ 4**	**TOTAL**	
**OBSERVER 1**	**Score ≤ 3**	15	2	17	k = 0.489
	**Score ≥ 4**	14	36	50	
**TOTAL**		29	38	67	
**SWIFTSCAN SPECT (OASIS)**		**OBSERVER 2**			
		**Score ≤ 3**	**Score ≥ 4**	**TOTAL**	
**OBSERVER 1**	**Score ≤ 3**	14	4	18	k = 0.722
	**Score ≥ 4**	1	17	18	
**TOTAL**		15	21	36	
**SIEMENS SPECT (OASIS)**		**OBSERVER 2**			
		**Score ≤ 3**	**Score ≥ 4**	**TOTAL**	
**OBSERVER 1**	**Score ≤ 3**	18	2	20	k = 0.775
	**Score ≥ 4**	2	14	16	
**TOTAL**		20	16	36	
**SWIFTSCAN SPECT (Manufacturer)**		**OBSERVER 2**			
		**Score ≤ 3**	**Score ≥ 4**	**TOTAL**	
**OBSERVER 1**	**Score ≤ 3**	13	2	15	k = 0.827
	**Score ≥ 4**	1	20	21	
**TOTAL**		14	22	36	
**SIEMENS SPECT (manufacturer)**		**OBSERVER 2**			
		**Score ≤ 3**	**Score ≥ 4**	**TOTAL**	
**OBSERVER 1**	**Score ≤ 3**	16	1	17	k = 0.889
	**Score ≥ 4**	1	18	19	
**TOTAL**		17	19	36	

## Discussion

In planar mode, GE LEHRS collimators associated to Clarity 2D provides higher image quality than the other collimators once there is diffusing material between the source and the camera.

In tomographic mode, the increase of volumetric sensitivity has reduced the noise in the images (low background variability) while maintaining at least an equivalent contrast recovery and spatial resolution.

On WBS, a better Siemens Symbia sensitivity can explain higher mean counts in bone ROIs and better absolute and relative contrasts than those observed with GE SwiftScan solution. On the other hand, GE SwiftScan reconstructions reduced noise with lower standard deviation in soft tissues ROIs. Noise reduction was responsible for a non-significant difference of contrast-to-noise ratios between both systems. This last parameter can be considered as closer to lesion contrast in pathological exams. This can explain why observers generally preferred GE SwiftScan image quality.

For planar acquisitions, we were expecting a loss of resolution with GE SwiftScan solution because of sensitivity improvement. In fact, the phantom study confirmed that resolution was lower than Siemens and GE LEHR systems when measured on detector surface. On the other hand, resolution recovery was more efficient with SwiftScan Clarity 2D so that resolution was better from 5 cm distance between source and collimator, which is often the case in clinical use. The superior resolution as compared with Siemens LEHR system is higher in PMMA attenuation conditions, which are closer to patient attenuation conditions. On clinical images, as seen on Fig 7, image quality is better with GE Swiftscan solution even on planar acquisitions in contact with the collimator.

All observers preferred SwiftScan planar image quality because of a better lesion delineation and less noise in soft tissues. This preference is not observed for all observers on WBS and might be due to faster scan on WBS which promotes sensitivity to resolution.

SPECT acquisitions are an important diagnostic added value in clinical use **[8]**. In this mode, we didn’t notice statistical difference between resolution and image quality between both systems. With step and shoot continuous mode, GE LEHRS collimators increased sensitivity of nearly 25%, compared to “classical” GE LEHR, and put the sensitivity just above the Siemens LEHR system. SwiftScan Clarity 2D image enhancement is not available in SPECT mode so we didn’t have the same processing for resolution recovery or noise optimization that could change the system performances **[9]**. This resulted in an equivalent image quality for all observers.

It could have been interesting to compare SPECT acquisitions between GE LEHR and SwiftScan LEHRS collimators in clinical use but the availability of only one NM670 camera in our department made this kind of clinical study difficult because of the time needed to change these collimators. This is why we compared this system with Siemens Symbia model as reference. The difference of image quality would have been likely more important. That would also have made possible to overcome manufacturer differences that can interfere in reconstruction process even if the impact is probably low **[10]**. This is the reason why we chose not to do attenuation and scatter corrections, and also why we reconstructed SPECT data with an independent software, to get rid of any manufacturer “black-box”.

## Conclusions

New GE SwiftScan collimator design improved sensitivity compared to “classical” GE LEHR collimator without compromising resolution.

Although slightly behind Siemens Symbia sensitivity, GE SwiftScan solution produced diagnostic quality images and improved planar image quality with a better Clarity 2D resolution recovery and noise treatment, compared to Symbia. Compared to Siemens, image quality was judged better for planar (all observers) and whole body scan (1 observer) images.

On SPECT mode, GE SwiftScan collimator with step and shoot continuous acquisitions improved volumetric sensitivity just over Siemens Symbia values but had no statistical impact on image quality.

This planar and SPECT sensitivity improvement also offers the perspective of shorter and more comfortable exams, especially in painful patients, or dose reduction.

## Supporting information

S1 File xls file of research data. This file contains all ROIs measurements and image scoring.
(XLSX)

S2 File Word file of study plan in French. (DOCX)

S3 File Pdf file of study plan in English. (PDF)

S4 File Word file of clinical trial form. (DOCX)

S5 File Pdf trend statement checklist. (PDF)
