# Supplementary material for: Clinical evaluation of General Electric new Swiftscan solution in bone scintigraphy on NaI-camera: A head to head comparison with Siemens Symbia
Source: PLoS One. 2019 Sep 19;14(9):e0222490. doi: 10.1371/journal.pone.0222490 (PMC6752842; doi:10.1371/journal.pone.0222490)

Étude clinique impliquant la personne humaine et ne comportant que des risques et contraintes minimes (catégorie 2 du L1121-1)

**LEHRS**

**Comparaison d’un nouveau collimateur LEHRS (General Electric – GE Healthcare) vs Siemens LEHR sur la qualité d’image en scintigraphie**

Version n°1.0 – 01/02/2018

| Promoteur | | | | |
| --- | --- | --- | --- | --- |
| **Centre Hospitalier Régional d’Orléans** Direction des affaires médicales et de la recherche 14 avenue de l’hôpital  CS 86709 45067 Orléans Cedex 02 | | |  | |
| Investigateur principal | | | | |
| Dr METRARD Gilles Service de médecine nucléaire – CHR d’Orléans 14, avenue de l’hôpital 45067 Orléans cedex 2 | | |  | |
| Protocole d’essai clinique | | | | |
| Code de l’étude | | | | CHRO 2018-03 |
| N° IDRCB | | | |  |
| AUTORISATIONS | | | | |
| Comité de Protection des Personnes | | | | |
| CPP | Numéro dossier : | Date de l'avis favorable : | | |
| Autorité Compétente | | | | |
| ANSM | Date de transmission pour information: | | | |
| Commission Nationale Informatique et Liberté | | | | |
| Déclaration de conformité à la méthodologie de référence (MR-001) : 17 mai 2013 | | | | |

CE DOCUMENT CONFIDENTIEL EST LA PROPRIETE DU CHR d’ORLEANS AUCUNE INFORMATION NON PUBLIEE FIGURANT DANS CE DOCUMENT NE PEUT ETRE DIVULGUEE SANS AUTORISATION ECRITE PREALABLE DU CENTRE HOSPITALIER.

Table des matières

[Résumé 6](#_Toc505606777)

[1. Informations générales 9](#_Toc505606778)

[1.1. Responsables du projet 9](#_Toc505606779)

[2. Contexte et justification scientifique 11](#_Toc505606784)

[2.1. Description de la pathologie 12](#_Toc505606785)

[2.2. Description de la stratégie étudiée 13](#_Toc505606786)

[2.3. Résumé des résultats des essais non cliniques et des essais cliniques disponibles 15](#_Toc505606787)

[3. Objectifs de la recherche 16](#_Toc505606788)

[3.1. Objectif principal 16](#_Toc505606789)

[3.2. Objectifs secondaires 16](#_Toc505606790)

[3.3. Critère d’évaluation principal 16](#_Toc505606791)

[3.4. Critères d’évaluation secondaires 16](#_Toc505606792)

[4. Population de l’étude 17](#_Toc505606793)

[4.1. Critères d’inclusion 17](#_Toc505606794)

[4.2. Critères de non-inclusion 17](#_Toc505606795)

[4.3. Modalités de recrutement 17](#_Toc505606796)

[4.4. Bénéfice / risque 18](#_Toc505606797)

[5. Conception de la recherche 18](#_Toc505606798)

[5.1. Méthodologie de la recherche 18](#_Toc505606799)

[5.2. Procédures spécifiques à La recherche 18](#_Toc505606800)

[5.3. Déroulement de l’étude 19](#_Toc505606801)

[5.4. Durée de la recherche 19](#_Toc505606802)

[5.5. Mesure prises pour réduire et éviter les biais 20](#_Toc505606803)

[Données recueillies dans le cahier d’observation 20](#_Toc505606804)

[5.6. Description des règles d’arrêt définitif ou temporaire 21](#_Toc505606805)

[6. Produits administrés aux personnes qui se prêtent à la recherche 22](#_Toc505606806)

[6.1. Traitements autorisés 22](#_Toc505606807)

[6.2. Traitements non autorisés 22](#_Toc505606808)

[6.3. Traitement d’urgence 22](#_Toc505606809)

[7. Evaluation de la sécurité 22](#_Toc505606810)

[7.1. Procédures mises en place en vue de l’enregistrement et de la notification des événements indésirables 22](#_Toc505606812)

[8. Statistiques 23](#_Toc505606813)

[*8.1.* méthodes statistiques prévues 23](#_Toc505606814)

[8.2. Nombre prévu de personnes à inclure dans la recherche 24](#_Toc505606815)

[8.3. Degré de signification statistique prévu 24](#_Toc505606816)

[8.4. Critères statistiques d’arrêt de la recherche 24](#_Toc505606817)

[8.5. Méthode de prise en compte des données manquantes, inutilisées ou non valides 24](#_Toc505606818)

[8.6. Choix des personnes à inclure dans les analyses 24](#_Toc505606819)

[9. Droit d’accès aux données et documents source 24](#_Toc505606820)

[9.1. Accès aux données 24](#_Toc505606821)

[9.2. Documents source 24](#_Toc505606822)

[9.3. Confidentialité des données 25](#_Toc505606823)

[10. Contrôle et assurance de la qualité 25](#_Toc505606824)

[11. Considérations éthiques 26](#_Toc505606825)

[11.1. Comité de Protection des Personnes 26](#_Toc505606826)

[11.2. Modifications substantielles 26](#_Toc505606827)

[11.3. Information du patient et consentement exprès 26](#_Toc505606828)

[11.4. Inscription au fichier national des personnes se prêtant à une recherche biomédicale 27](#_Toc505606829)

[12. Produit des données et conservation des documents et données 27](#_Toc505606830)

[12.1. Fiche d’observation 27](#_Toc505606831)

[12.2. Saisie et produit des données 27](#_Toc505606832)

[12.3. CNIL 27](#_Toc505606833)

[12.4. Archivage 28](#_Toc505606834)

[13. Financement et assurance 28](#_Toc505606835)

[13.1. Budget de l’étude 28](#_Toc505606836)

[13.2. Assurance 29](#_Toc505606837)

[14. Faisabilité de l'étude 29](#_Toc505606838)

[15. Règles relatives à la publication 29](#_Toc505606839)

[16. Bibliographie 30](#_Toc505606840)

[17. Liste des annexes 30](#_Toc505606841)

# Accord du protocole

SIGNATURE DE L'INVESTIGATEUR

| J'ai lu l’ensemble des pages du protocole de l’essai clinique dont le CHR d’ORLEANS est le promoteur. Je confirme qu'il contient toutes les informations nécessaires à la conduite de l’essai. Je m'engage à réaliser l’essai en respectant le protocole et les termes et conditions qui y sont définis. Je m'engage à réaliser l’essai en respectant :   - les principes de la “Déclaration d’Helsinki”, - les règles et recommandations de bonnes pratiques cliniques internationales (ICH) et française (règles de bonnes pratiques cliniques pour les recherches biomédicales portant sur des médicaments à usage humain) - la législation nationale et la réglementation relative aux essais cliniques, - la conformité avec la Directive Essais Cliniques de l’UE [2001/20/EC].   Je m'engage également à ce que les investigateurs et les autres membres qualifiés de mon équipe aient accès aux copies de ce protocole et des documents relatifs à la conduite de l’essai pour leur permettre de travailler dans le respect des dispositions figurant dans ces documents. |
| --- |
| **NOM :**  Signature : Date : ___________________  *Conformément aux dispositions de la loi relative à l’informatique, aux fichiers et aux libertés, vous disposez à tout moment d’un droit d’accès et de rectification des données informatisées vous concernant (loi n° 2004-801 du 6 août 2004 modifiant la loi n° 78-17 du 6 janvier 1978 relative à l’informatique, aux fichiers et aux libertés).* |

SIGNATURE DU PROMOTEUR

| **Promoteur :** | |
| --- | --- |
| NOM : Antoine LEBRERE  Directeur des affaires médicales et de la Recherche  Signature : | Date : ___________________ |

# Résumé

| Titre | Comparaison d’un nouveau collimateur GE LEHRS vs Siemens LEHR sur la qualité d’image en scintigraphie. |
| --- | --- |
| Acronyme | LEHRS |
| Promoteur | **Centre Hospitalier Régional d’Orléans** Direction des affaires médicales et de la recherche 14 avenue de l’hôpital  45067 Orléans Cedex 02 |
| Investigateur principal | **Dr Gilles METRARD**  **Centre Hospitalier Régional d’Orléans**  Service de médecine nucléaire 14 avenue de l’hôpital  45067 Orléans Cedex 02 |
| Version du protocole | V1.0 du 1er février 2018 |
| Justification / contexte | La réalisation d’un examen scintigraphique monophotonique avec une gamma-caméra nécessite l’utilisation obligatoire d’un collimateur. Cette grille perforée fait l’objet d’études industrielles à la recherche du meilleur compromis entre, d’un côté, la taille et le nombre de « trous » (déterminant la sensibilité) et, d’un autre côté, l’épaisseur et la longueur des cloisons (conditionnant la résolution). Ces paramètres sont inversement liés car l’augmentation des surfaces ouvertes diminue d’autant la surface des cloisons. |
| Objectif Principal | Comparer la qualité d’image obtenue avec le nouveau collimateur LEHRS GE à celle obtenue avec un collimateur standard LEHR Siemens dans les examens de scintigraphie osseuse. |
| Objectifs Secondaires | Evaluation de la qualité d’image dans les scintigraphies pulmonaires, thyroïdiennes, parathyroïdiennes et cérébrales au DaTSCAN.  Evaluation des performances sur des objets-tests de mesures physiques. |
| Critère de Jugement Principal | Taux d’images obtenant un score supérieur ou égal à 4 en analyse visuelle de la qualité d’image sur une échelle de type 5 points LIKERT. |
| Critères de Jugement Secondaires | Mesures de rapports signal sur bruit sur les acquisitions patients.  Calcul des performances physiques du nouveau collimateur LEHRS à l’aide d’objets-tests de mesures physiques. |
| Méthodologie / Schéma de l’etude | Etude monocentrique, comparative, de non infériorité, avec comparaisons intra-patients |
| Critères d’Inclusion des Sujets | Patients externes adressés dans le service pour une scintigraphie osseuse, pulmonaire, thyroïdienne, parathyroïdienne ou cérébrale au DaTSCAN. |
| Critères de Non-Inclusion des Sujets | Enfants, femmes enceintes ou à risque de grossesse, Patients sous tutelle ou curatelle, insuffisance rénale connue, patients algiques, patients ne pouvant bénéficier d’un examen scintigraphique standard de bonne qualité. |
| Stratégies / Procédures | La scintigraphie des patients sera réalisée sur 2 appareils distincts et les images obtenues seront comparées. |
| Nombre de Patients | 85 patients dont 70 pour scintigraphie osseuse |
| Durée de la Recherche | Durée de la période d’inclusion : 1 an  Durée de la participation pour chaque patient : inférieure à une demi-journée.  Durée totale de l’étude : 12 mois (inclusion et analyse des données) |
| Retombées attendues | A titre individuel pour le patient : vraisemblable amélioration de la qualité d’image de son examen, relecture de l’examen par 2 médecins experts.  A titre collectif : amélioration de la qualité d’image des examens scintigraphiques avec ce collimateur permettant de réduire la durée d’examen et/ou l’activité injectée de médicament radio-pharmaceutique (amélioration de la dosimétrie patient). |

Liste des abréviations

Tableau à compléter selon le contenu du protocole ci-après

| ANSM | Agence nationale de sécurité du médicament et des produits de santé |
| --- | --- |
| AMM | Autorisation de Mise sur le Marché |
| ARC | Attaché de Recherche Clinique |
| BPC | Bonnes Pratiques Cliniques |
| CPP | Comité de Protection des Personnes |
| CNIL | Commission Nationale de l’Informatique et des Libertés |
| CRF | Case Report Form (cahier d’observation) |
| EvIG | Evènement Indésirable Grave |
| EIG | Effet Indésirable Grave |
| EIGI | Effet Indésirable Grave Inattendu |
| GE | General Electric Healthcare |
| ICH | International Conference on Harmonization (Conférence internationale pour l'harmonisation) |
| IDE | Infirmière Diplômée d'Etat |
| INSERM | Institut National de la Santé et de la Recherche Médicale |
| LEHR | Low Energy High Resolution : basse énergie haute résolution |
| MR | Méthodologie de Référence |
| RCP | Résumé des Caractéristiques d'un Produit |
| SUSAR | Suspected Unexpected Serious Adverse Reaction |
| TEC | Technicien d'Etude Clinique |
| TDM | Tomodensitométrie |

# Informations générales

## Responsables du projet

| Titre de l’étude : LEHRS  Comparaison d’un nouveau collimateur LEHRS (General Electric – GE Healthcare) vs Siemens LEHR sur la qualité d’image en scintigraphie | |
| --- | --- |
| **Promoteur :** | CHR D’Orléans  Direction des affaires médicales et de la recherche 14 avenue de l’hôpital  CS 86709 45067 Orléans Cedex 02 |
| **Investigateur coordonnateur :** | **Dr Gilles METRARD**  Service de Médecine Nucléaire  CHR Orléans |
| **Investigateurs associés :** | Dr Matthieu BAILLY  Dr Hélène BESSE  Dr Sabine GAUVAIN  Dr Sofiane MOUZOUNE |
| **Methodologiste / Biostatisticien :** | **Dr Antoine VALERY**  DIM  CHR Orléans |

# Contexte et justification scientifique

Les examens scintigraphiques permettent de cartographier, in vivo, la biodistribution d'un médicament radiopharmaceutique, analogue d’une molécule d'intérêt.

L’image de la distribution dans l'organisme du médicament radiopharmaceutique est obtenue par la détection des rayonnements gamma émis par le radioélément utilisé pour son marquage.

L'adjonction d'un collimateur (grille perforée en plomb) devant les détecteurs est obligatoire afin de sélectionner les photons gamma, les plus informatifs, dont le trajet est orthogonal au plan des détecteurs.

Le choix du collimateur utilisé repose sur un compromis entre deux paramètres :

- L'épaisseur et la profondeur des cloisons. Plus l'énergie du photon gamma émis (spécifique de chaque radioélément) est importante, plus le photon a une probabilité élevée de traverser la matière, notamment les cloisons (septas) du collimateur. Cette pénétration septale entraîne une imprécision sur l'origine du rayonnement qui se traduit par une altération de la résolution spatiale, donc de la qualité d'image. Pour cette raison, les collimateurs sont classés en trois grandes catégories, selon l’épaisseur croissante de leurs septas : basse énergie (« low energy », LE), moyenne énergie (« mean energy » ou ME) et haute énergie (« high energy » HE).
- La largeur et le nombre des trous entre les cloisons. De larges trous permettent d'augmenter la sensibilité de la gamma-caméra mais réduisent, à surface constante de détecteurs, le nombre ou l'épaisseur des cloisons. La sensibilité permet donc de réduire le temps d’examen, au prix d’une altération de la résolution.

La majorité des examens de médecine nucléaire utilisent des médicaments radiopharmaceutiques technétiés (marqués au technetium 99m, émetteur de photons gamma, de pic énergétique 140keV) et nécessitent l’utilisation de collimateurs basse énergie (LE). Il existe de grandes versions de ce type de collimateur, la première privilégiant la sensibilité et la seconde privilégiant la résolution (LEHR pour « Low Energy High Resolution »).

Nous proposons donc d'évaluer un nouveau type intermédiaire de collimateurs (LEHRS pour « Low Energy High Resolution and Sensitivity », GE) privilégiant la sensibilité, sans cependant sacrifier la résolution. La dégradation attendue de la résolution étant compensée par des algorithmes correctifs informatiques.

## Description de la pathologie

La scintigraphie osseuse est un examen de pratique courante en médecine nucléaire. Le médicament radiopharmaceutique utilisé dans cette étude est un biphosphonate marqué au technétium 99m (dans notre service, il s’agit de l’hydroxydiphosphonate ou HDP-99mTc).

C’est un examen qui quantifie, de façon sensible, l’anabolisme osseux en réponse à des pathologies et permet de visualiser l’ensemble du squelette en un seul examen.

Les pathologies touchant l’os sont multiples et peuvent être d’origine tumorale, traumatique, rhumatismale ou infectieuse. Ainsi les indications de la scintigraphie osseuse sont variées : rhumatologiques et orthopédiques (exploration de douleurs osseuses diffuses, bilan de rhumatisme inflammatoire, datation de fractures, recherche d’algoneurodystrophie, exploration de douleurs sur prothèse, notamment recherche de descellement…), oncologiques (bilan d’extension de tumeurs osseuses, notamment des tumeurs à métastases ostéocondensantes comme dans les cancers de prostate, bilan d’extension des sarcomes et autres tumeurs osseuses…)

Les principaux avantages de la scintigraphie osseuse reposent sur une bonne accessibilité de la technique sur l’ensemble du territoire (à la différence de l’IRM), son exploration corps entier, en un temps d’examen réduit (balayage corps entier en environ 15 minutes), pour une irradiation relativement faible comparativement à l’apport diagnostique de la technique. Pour un adulte de 70kg, la dose efficace est de 4mSv ; à titre comparatif, l’irradiation naturelle à Orléans (liée au rayonnement cosmique et tellurique des sols) est d’environ 1 à 2mSv par an. Un scanner thoraco-abdomino-pelvien délivre quant à lui en moyenne une dose efficace de 18mSv selon le rapport de l’IRSN de 2010 « Doses délivrées aux patients en scanographie et en radiologie conventionnelle ».

Il est également important de préciser qu’à la différence des examens de radiologie, l’irradiation liée à une scintigraphie est liée à la posologie du médicament radiopharmaceutique et n’augmente donc pas avec le nombre d’acquisitions scintigraphiques réalisées.

## Description de la stratégie étudiée

Les patients bénéficieront de leur examen de scintigraphie standard, selon les règles de bonnes pratiques éditées par les sociétés savantes françaises et internationales. Ainsi, selon l’indication de leur examen, des images précoces au décours de l’injection du médicament radiopharmaceutique seront réalisées. Il sera en revanche systématiquement réalisé des clichés tardifs de scintigraphie osseuse, 2 heures après injection (pour le médicament radiopharmaceutique utilisé dans le service), à savoir un balayage corps entier du squelette. Cette acquisition est souvent complétée par des clichés statiques, centrés sur une zone du squelette (le plus souvent la zone douloureuse et/ou présentant une hyperfixation pathologique du radiopharmaceutique).

Dans certains cas, une tomoscintigraphie sera réalisée, permettant d’obtenir des images de scintigraphie en coupe, dans les 3 plans de l’espace, améliorant la sensibilité de détection de petites fixations osseuses anormales, et surtout la localisation de ces foyers. Cette acquisition de tomoscintigraphie sera couplée à des coupes de tomodensitométrie (TDM) permettant une localisation anatomique encore plus précise.

Outre cet examen standard, la participation du patient au protocole de recherche impliquera la réalisation d’images complémentaires avec le nouveau collimateur, à savoir un deuxième balayage corps entier, un cliché statique, et dans les cas où elle a été demandée pour l’examen standard, une tomoscintigraphie (sans couplage à la TDM dans ce cas, pour maintenir une dosimétrie équivalente à l’examen standard).

## Résumé des résultats des essais non cliniques et des essais cliniques disponibles

Aucune étude n’a encore été réalisée pour évaluer ce nouveau collimateur.

Peu de données de la littérature sont disponibles quant à l’évaluation de nouveaux collimateurs en médecine nucléaire. La plupart des études sont des études de physique médicale, visant à évaluer les performances physiques en termes de sensibilité et de résolution sur objets de mesures physiques (par exemple, l’évaluation d’un nouveau collimateur en tomosynthèse mammaire, Gilland et al. Med Phys 2017 Nov;44(11):5740-5748). Une évaluation sur objets de mesures physiques de l’ancien et du nouveau collimateur est prévue dans notre étude.

Notre travail de recherche peut être mis en parallèle des études réalisées suite à la commercialisation des caméras de médecine nucléaire à semi-conducteurs (dites CZT, pour cadmium-zinc-telluride). Par exemple, dans l’étude rétrospective menée par l’équipe du CCN (Songy et al, [Clin Nucl Med.](https://www.ncbi.nlm.nih.gov/pubmed/21825848) 2011 Sep;36(9):776-80), 153 patients avaient bénéficié de leur scintigraphie cardiaque sur une caméra d’ancienne génération, puis sur la nouvelle caméra CZT. Les données étaient analysées de façon rétrospective, avec comparaison de la qualité d’image et aussi du diagnostic final retenu.

# Objectifs de la recherche

## Objectif principal

Comparer la qualité d’image obtenue avec le nouveau collimateur LEHRS GE à celle obtenue avec un collimateur standard LEHR Siemens dans les examens de scintigraphie osseuse.

## Objectifs secondaires

Evaluation des performances sur des objets-tests de mesures physiques.

Evaluation de la qualité d’image dans les scintigraphies pulmonaires, thyroïdiennes, parathyroïdiennes et cérébrales au DaTSCAN.

## Critère d’évaluation principal

Le critère d’évaluation principal sera l’évaluation de la qualité d’image en scintigraphie osseuse obtenue avec le nouveau collimateur LEHRS GE vs celle obtenue avec un collimateur standard LEHR Siemens, par deux médecins nucléaires experts.

Il n'existe pas de critère standardisé d'évaluation de la qualité d'image en scintigraphie. Une image scintigraphique de bonne qualité (sensible et avec une bonne résolution spatiale) présentera une fixation osseuse de qualité, avec un faible bruit de fond, des surfaces osseuses bien définies, ainsi qu'une bonne visualisation des espaces interosseux.

Nous utiliserons donc une échelle en 5 points, type Likert :

5 Qualité diagnostique – Excellente qualité d’image et résolution (excellente fixation osseuse, bruit de fond quasi-inexistant, bonne visualisation des surfaces osseuses et des espaces interosseux)

4 Qualité diagnostique – Bonne qualité d’image et résolution (bonne fixation osseuse, bruit de fond faible, bonne visualisation des surfaces osseuses et des espaces interosseux)

3 Qualité diagnostique –Qualité d’image et résolution acceptables (bonne fixation osseuse, bruit de fond modéré, visualisation plus floue des surfaces osseuses et des espaces interosseux)

2 Qualité sous-optimale – Information clinique limitée (faible fixation osseuse, bruit de fond modéré voire important, mauvaise visualisation des surfaces osseuses et des espaces interosseux)

1. Qualité non diagnostique (mauvaise fixation osseuse voire quasi-absence de fixation osseuse, bruit de fond important)

Pour chaque patient, ce score sera établi pour chaque type d'image et pour chaque collimateur, à savoir le balayage corps entier, un cliché statique, la tomoscintigraphie (si réalisée).

Le pourcentage d’examens jugés de bonne qualité (scores 4 et 5), sera comparé pour chaque collimateur et chaque type d’image.

## Critères d’évaluation secondaires

Les critères d’évaluation secondaires seront :

- Des mesures physiques de performance des collimateurs sur objet-test de mesure.
- Une évaluation du rapport signal/bruit dans les images de scintigraphie osseuse (ratios de fixation sacro-iliaque / tissus mous sur le balayage corps entier) obtenues à l’aide des deux jeux de collimateurs.
- Une évaluation quantifiée (échelle de Likert) de la qualité d’image obtenue dans les examens scintigraphiques pulmonaires, thyroïdiens, parathyroïdiens ou cérébraux au DaTSCAN.
- Une évaluation du rapport signal/bruit dans les images de scintigraphie cérébrale au DaTSCAN, en comparant les ratios de fixation striata/occiput obtenus à l’aide des deux jeux de collimateurs, après analyse et segmentation automatique dans le logiciel DaTQUANT (GE).

# Population de l’étude

## Critères d’inclusion

Tout patient adressé pour une scintigraphie osseuse, pulmonaire, thyroïdienne, parathyroïdienne ou cérébrale au DaTSCAN.

## Critères de non-inclusion

- Mineurs,
- Femmes enceintes ou à risque de grossesse (un dosage plasmatique de béta-HCG sera réalisé dans les 48h précédant l’examen pour les patientes en seconde partie de cycle, en l’absence de contraception efficace),
- Patients souffrant d’insuffisance rénale connue,
- Patients algiques, ayant une EVA >4,
- Patients ne pouvant bénéficier d’un examen scintigraphique standard de bonne qualité (patients agités …)
- Personne sous tutelle ou sous curatelle

## Modalités de recrutement

Tout patient externe ou hospitalisé « valide », adressé pour une scintigraphie osseuse, pulmonaire, thyroïdienne, parathyroïdienne ou cérébrale au DaTSCAN, recevra avec sa convocation la notice d'information de l'étude.

Le patient sera vu par un médecin nucléaire de l’équipe dès son arrivée dans le service de médecine nucléaire, avant sa prise en charge. Le protocole de l’étude et les différentes informations lui seront alors expliqués.

Les documents informatifs seront laissés au patient qui pourra continuer d’en prendre connaissance, de manière plus approfondie, pendant le temps d'attente avant la réalisation des acquisitions de l'examen (environ 2 heures après l’injection du médicament radiopharmaceutique).

Si au terme de cette période de réflexion additionnelle, le patient accepte toujours de participer l'étude, sa fiche de consentement sera recueillie par le manipulateur, qui la classera dans le dossier de l'étude. En cas de refus, l’examen standard sera réalisé et le patient ne participera pas à l’étude.

## Bénéfice / risque

La participation à cette étude n'engendre aucun risque supplémentaire pour le patient, puisque la réalisation de la 2^ème^ scintigraphie n’entraine pas de modification de la posologie, du mode et de la fréquence (injection unique) d’administration du médicament radiopharmaceutique injecté.

Le patient bénéficiera d’une vraisemblable amélioration de la qualité d’image de son examen et d’une relecture de l’examen par 2 médecins experts. La seule contrainte pour le patient sera une durée d’examen allongée de 15 à 30 minutes, par rapport à un examen « standard », sans autre acte invasif ou irradiation supplémentaire.

A titre collectif, l’amélioration de la qualité d’image des examens scintigraphiques avec ce collimateur pourrait permettre de réduire la durée d’examen et/ou l’activité injectée de médicament radio-pharmaceutique (amélioration de la dosimétrie patient).

# Conception de la recherche

## Méthodologie de la recherche

Etude monocentrique, comparative, de non infériorité, avec comparaisons intra-patients (acquisition contrôle sur le collimateur de référence LEHR Siemens, pour chaque patient).

Interprétation de la qualité des images en double insu.

## Procédures spécifiques à La recherche

La prise en charge des patients inclus dans cette étude est calquée sur la prise en charge standard conventionnelle.

Il sera simplement réalisé, avec le médicament radiopharmaceutique déjà injecté pour l’examen standard, un nombre de clichés plus importants (réalisés avec le jeu de deux collimateurs à évaluer), mais sans irradiation complémentaire.

En cas de tomoscintigraphie couplée à la TDM réalisée pour l’examen standard (selon l’indication clinique de l’examen), seule une acquisition TDM sera réalisée pour les deux tomoscintigraphies avec des collimateurs différents.

## Déroulement de l’étude

### Inclusion

Les critères d’inclusion et de non inclusion seront vérifiés lors d’une consultation médicale avec le patient. Cette consultation sera réalisée dès l’arrivée dans le service ou, au plus tard, avant la réalisation des acquisitions tardives (environ 2h après l’injection de l’examen standard prescrit).

L’information et le recueil du consentement seront réalisés au cours de cette consultation avec un des médecins du service. Au cours de cette consultation, seront également vérifiés les différents critères d'inclusion et de non-inclusion.

Après un délai de réflexion, en cas de participation à cette étude, les différents documents seront colligés dans le dossier du patient, au plus tard avant la réalisation des clichés tardifs de l’examen standard de scintigraphie.

À tout moment, le patient pourra, s’il le manifeste, quitter l'étude. Dans ce cas, seuls des acquisitions nécessaires à l'examen standard seront réalisés.

### Suivi des patients

Aucun suivi des patients, autre que celui nécessaire pour la pathologie explorée en scintigraphie standard, ne sera nécessaire à l’étude.

## Durée de la recherche

La période d’inclusion se déroulera sur une durée de 12 mois après l’avis favorable du CPP pour la réalisation de l’étude et la mise à disposition du matériel (collimateur …).


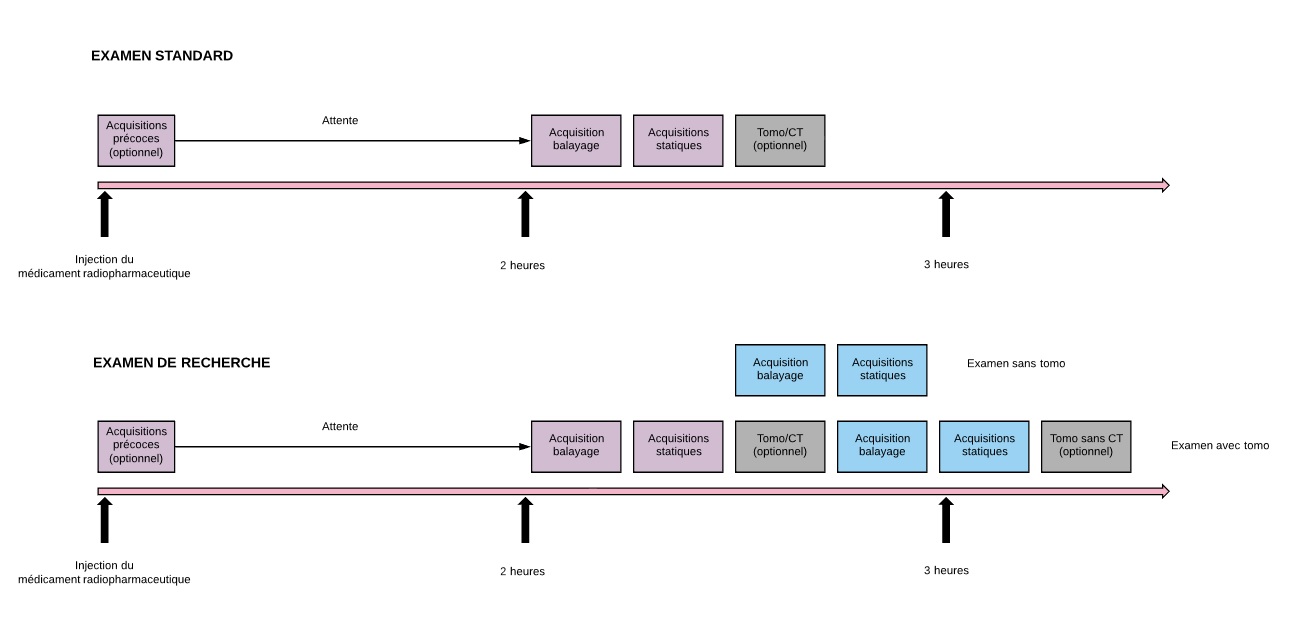
La durée totale de participation pour le patient ne dépassera pas la demi-journée.

## Mesure prises pour réduire et éviter les biais

### Tirage au sort

Il ne sera pas réalisé de tirage au sort car l’examen standard sera toujours réalisé en premier afin de ne pas entrainer de perte de chance si l’examen devait être arrêté (patient algique …). Ceci laisse également la possibilité au patient de refuser la participation à l’étude à la fin de son examen standard.

Le protocole sera adapté pour compenser le délai entre les deux examens (augmentation de la durée d’acquisition proportionnellement à la décroissance radioactive).

L’inclusion des patients se fera de façon consécutive. Le patient réalisera donc ses images en premier de façon aléatoire sur la caméra GE, équipée du collimateur LEHRS, ou sur la caméra Siemens, avec collimateur LEHR, en fonction de son créneau de rendez-vous initial. Cependant, lorsque 50% de l'effectif aura été réalisée sur une des deux caméras, les inclusions se poursuivront uniquement sur l'autre caméra.

Outre la correction de la décroissance radioactive sus-décrite, cette répartition permettra de s’affranchir de l’augmentation de captation osseuse du radiopharmaceutique avec le temps.

### Méthode de mise en insu

Les données d’imagerie seront anonymisées, par une personne qui ne fera pas partie des relecteur des images, pour faire disparaître, des champs DICOM de l’image (métadonnées), toutes les informations relatives à l’identité du patient. Un numéro d'anomalie limitation sera attribuée à chaque patient, au moyen d'une table de randomisation. La correspondance identité du patient – numéro d’anonymisation sera recueillie dans une base de données spécifique.

La lecture des images sera réalisée à la fin des inclusions, en insu, à partir des données anonymisées, par deux médecins séniors, sur console de post-traitement. La méthode d’anonymisation ne permettra pas d'identifier ni l’identité du patient, ni le type d'appareil utilisé (collimateur LEHR ou LEHRS).

## Données recueillies dans le cahier d’observation

- Données liées au patient : âge, sexe, poids, IMC.
- Données liées au médicament radiopharmaceutique : libellé du médicament radiopharmaceutique, activité injectée, heure et site d’injection.
- Données liées aux acquisitions : horaires d’acquisition, paramètres physiques (pic énergétique, fenêtrage, durée d’acquisition, temps par projection et nombre de projections si tomoscintigraphie…)

Comme pour l’examen standard, les données d’imagerie de l’étude seront stockées et archivées dans le PACS de l’établissement.

## Description des règles d’arrêt définitif ou temporaire

### Arrêt de participation d’une personne à la recherche

Les sujets pourront retirer leur consentement et demander à sortir de l’étude à n’importe quel moment et quelle qu’en soit la raison. En cas de sortie prématurée, l’investigateur doit en documenter les raisons de façon aussi complète que possible.

L’investigateur pourra interrompre temporairement ou définitivement la participation d’un sujet à l’étude pour toute raison qui servirait au mieux les intérêts du sujet en particulier en cas de symptomatologie douloureuse ou de claustrophobie majeure lors des acquisitions

La sortie d'étude d'un patient ne changera en rien sa prise en charge habituelle par rapport à sa maladie.

### Arrêt d’une partie ou de la totalité de la recherche

L’étude peut être interrompue prématurément en cas de survenue d’événements indésirables inattendus, graves nécessitant une revue du profil de la stratégie. De même, des événements imprévus ou de nouvelles informations relatives au produit, au vu desquels les objectifs de l'étude ou du programme clinique ne seront vraisemblablement pas atteints, peuvent amener le promoteur à interrompre prématurément l’étude.

Le CHR d’ORLEANS se réserve le droit d'interrompre l’étude, à tout moment, s'il s'avère que les objectifs d’inclusion ne sont pas atteints.

# Produits administrés aux personnes qui se prêtent à la recherche

Une injection de médicament radiopharmaceutique, nécessaire à la réalisation de l’examen scintigraphique standard, sera réalisée, comme dans la pratique courante. Il n’y aura pas d’injection supplémentaire en lien avec le protocole de recherche. Les résumés des caractéristiques des produits (RCP) des médicaments radiopharmaceutiques utilisés dans cette étude sont présentés en annexe.

## Traitements autorisés

Traitement habituels du patient, antalgiques…

## Traitements non autorisés

Aucun traitement n’est concerné. Tout traitement non autorisé pour l’examen standard entrainera son annulation et, de fait, la participation à l’étude (vérification des dernières dates de chimiothérapie, injection de biphosphonates, anti-thyroïdiens de synthèse et hormones thyroïdiennes…)

## Traitement d’urgence

Non concerné. Aucun effet indésirable majeur n’est documenté pour les médicaments radiopharmaceutiques utilisés lors de l’examen standard.

# Evaluation de la sécurité

## Procédures mises en place en vue de l’enregistrement et de la notification des événements indésirables

S’agissant d’une étude de catégorie 2, aucune vigilance particulière liée au protocole de recherche ne sera mise en place.

Cependant, la surveillance et le signalement des incidents et risques d’incidents résultant de la participation à l’étude seront déclarés via le circuit habituel. Le collimateur et les logiciels utilisés étant marqués CE, les évènements indésirables éventuels feront l’objet d’une déclaration de matériovigilance par le CHR d’ORLEANS.

## Comité de Surveillance

La participation à l’étude n’entrainant aucun risque pour le patient, il n’a pas été jugé nécessaire de mettre en place un comité de surveillance.

## Modalités et durée du suivi des personnes suite à la survenue d’événements indésirables

Chaque évènement indésirable sera suivi jusqu’à sa complète résolution (stabilisation à un niveau jugé acceptable par l’investigateur ou retour à l’état antérieur) même si le patient est sorti d’essai.

# Statistiques

**Biostatisticien**

Dr Antoine Valéry (CHR d’Orléans)

**Logiciels utilisés**

- R Core Team (2017). R: A language and environment for statistical computing. R Foundation for Statistical Computing, Vienna, Austria. URL <https://www.R-project.org/>
- Version 1.1.423 – © 2009-2018 RStudio, Inc.

## méthodes statistiques prévues

### Statistiques descriptives et constitution du jeu de données

Pour chaque variable continue une analyse préalable de la distribution permettra de déterminer s’il y a lieu de procéder à une transformation de variable.

Les variables continues seront décrites par la moyenne et l’écart type, les variables ordinales, par la médiane et l’intervalle interquartile.

Les variables booléennes ou discrètes nominales seront présentées

- En effectif
- En proportions sous forme de pourcentage + intervalle de confiance à 95%
- Graphiquement par histogramme pour représentation en mosaïque

### Statistiques inférentielles

Compte tenu de la taille de l’échantillon à l’étude on utilisera des méthodes paramétriques pour les statistiques en population entière.

#### Concordance des évaluations

La concordance du score diagnostic entre observateurs sera évaluée par :

1. Calcul des coefficients de corrélation intra-classe
2. Analyse graphique de Bland et Altman

#### Objectif principal

La recherche préalable d’une relation entre la qualité de l‘image et l’IMC ou l’âge des patients sera conduite au moyen d’une comparaison des moyennes de ces variables en fonction du score diagnostic (<4 versus ≥4) pour chacun des deux collimateurs.

La différence de qualité diagnostique entre les deux dispositifs sera explorée par comparaison appariée des pourcentages d’image obtenant un score supérieur ou égal à 4 au moyen du test de Mac Nemar.

Au cas où un ou plusieurs facteurs de confusion seraient identifiés, une analyse ajustée par la méthode de Mantel-Haenszel  sera réalisée.

#### Objectifs secondaires

Les comparaisons de performance des dispositifs conduites en sous-groupes (topographie d’examen ou objets test) feront appel au test de Mac Nemar.

Comme pour l’objectif principal, si un ou plusieurs facteurs de confusion sont identifiés, une analyse ajustée par la méthode de Mantel-Haenszel sera réalisée.

## Nombre prévu de personnes à inclure dans la recherche

Sur la base des performances estimées connues du collimateur de référence, le calcul du nombre de sujets nécessaire repose sur une hypothèse de non infériorité du nouveau dispositif à la marge de non infériorité de 12 %.

Le recueil en un seul temps des données ne justifie pas de prévoir un taux de perdus de vue.

- p1 : % d’images de bonne qualité attendu avec collimateur de référence = 97%
- p2 : % d’images de bonne qualité attendu avec le nouveau collimateur = 85%
- Marge de non-infériorité p2-p1 = 12%
- α Risque de première espèce = 5% 1-β Puissance = 80% Formulation : unilatérale

**Méthode  de calcul :** package epiR fonction epi.prosize

**n Nombre de sujets nécessaire = 70**

## Degré de signification statistique prévu

Pour l’ensemble des tests statistiques, le risque de première espèce est fixé à α = 5%.

## Critères statistiques d’arrêt de la recherche

Aucun.

## Méthode de prise en compte des données manquantes, inutilisées ou non valides

Les patients pour lesquels un critère de jugement principal est manquant seront exclus des analyses.

# Droit d’accès aux données et documents source

## Accès aux données

Conformément aux BPC :

- le promoteur est chargé d’obtenir l’accord de l’ensemble des parties impliquées dans la recherche afin de garantir l’accès direct à tous les lieux de déroulement de la recherche, aux données source, aux documents source et aux rapports dans un but de contrôle de qualité et d’audit par le promoteur,

- les investigateurs mettront à disposition des personnes chargées du suivi, du contrôle de qualité ou de l'audit de la recherche biomédicale, les documents et données individuelles strictement nécessaires à ce contrôle, conformément aux dispositions législatives et réglementaires en vigueur (articles L.1121-3 et R.5121-13 du code de la santé publique).

## Documents source

Les documents source étant définis comme tout document ou objet original permettant de prouver l'existence ou l'exactitude d'une donnée ou d'un fait enregistrés au cours de l’[étude clinique](http://134.157.220.13/urcest/sous_cadre.php?fich=Lexique/E.htm#essaiclinique) seront conservés pendant 25 ans par l'investigateur ou par l'hôpital s'il s'agit d'un dossier médical hospitalier.

## Confidentialité des données

Conformément aux dispositions concernant la confidentialité des données auxquelles ont accès les personnes chargées du contrôle de qualité d’une recherche biomédicale (article L.1121-3 du code de la santé publique), conformément aux dispositions relatives à la confidentialité des informations concernant notamment la nature des produits , les essais, les personnes qui s'y prêtent et les résultats obtenus (article R. 5121-13 du code de la santé publique), les personnes ayant un accès direct prendront toutes les précautions nécessaires en vue d'assurer la confidentialité des informations relatives aux produits , aux essais, aux personnes qui s'y prêtent et notamment en ce qui concerne leur identité ainsi qu’aux résultats obtenus.

Ces personnes, au même titre que les investigateurs eux-mêmes, sont soumises au secret professionnel (selon les conditions définies par les articles 226-13 et 226-14 du code pénal).

Pendant la recherche biomédicale ou à son issue, les données recueillies sur les personnes qui s’y prêtent et transmises au promoteur par les investigateurs (ou tous autres intervenants spécialisés) seront rendues anonymes.

Elles ne doivent en aucun cas faire apparaître en clair les noms des personnes concernées ni leur adresse.

Seules la première lettre du nom du sujet et la première lettre de son prénom seront enregistrées, accompagnées d’un numéro codé propre à l’étude indiquant l’ordre d’inclusion des sujets. Les images du patient seront anonymisées, au moyen d'une fonctionnalité spécifique de la console de traitement d'image.

Le promoteur s’assurera que chaque personne qui se prête à la recherche a donné son accord par écrit pour l’accès aux données individuelles la concernant et strictement nécessaires au contrôle de qualité de la recherche.

# Contrôle et assurance de la qualité

Un Attaché de Recherche Clinique (ARC) mandaté par le promoteur s’assurera de la bonne réalisation de l’étude.

L'investigateur et les membres de son équipe acceptent de se rendre disponibles lors des visites de Contrôle de Qualité effectuées à intervalles réguliers par l’Attaché de Recherche Clinique. Lors de ces visites, les éléments suivant seront revus :

- consentement exprès
- respect du protocole de l'étude et des procédures qui y sont définies

D’autre part, Les investigateurs s’engagent à accepter les audits d’assurance qualité effectués par le promoteur ainsi que les inspections effectuées par les Autorités Compétentes. Toutes les données, tous les documents et rapports peuvent faire l'objet d'audits et d'inspections réglementaires sans que puisse être opposé le secret médical.

# Considérations éthiques

## Comité de Protection des Personnes

Le protocole, le formulaire d'information de l'étude seront soumis pour avis à un Comité de Protection des Personnes désigné par tirage au sort.

La notification de l'avis favorable du CPP sera transmise au promoteur de l'étude et à l'Autorité compétente.

## Modifications substantielles

En cas de modification substantielle apportée au protocole par l’investigateur, elle sera approuvée par le promoteur. Ce dernier devra obtenir préalablement à sa mise en œuvre un avis favorable du CPP. Un nouveau consentement des personnes participant à la recherche sera recueilli si nécessaire.

## Information du patient et consentement exprès

Les patients seront informés de façon complète et loyale, en des termes compréhensibles, des objectifs et des contraintes de l'étude, des risques éventuels encourus, des mesures de surveillance et de sécurité nécessaires, de leurs droits de refuser de participer à l'étude ou de la possibilité de se rétracter à tout moment.

Toutes ces informations figurent sur un formulaire d’information remis au patient. Le consentement exprès et écrit du patient sera recueilli par l’investigateur, ou un médecin qui le représente avant l’inclusion définitive dans l’étude.

## Inscription au fichier national des personnes se prêtant à une recherche biomédicale

Cette inscription n’est pas prévue dans le cadre de la recherche

# Produit des données et conservation des documents et données

## Fiche d’observation

Toutes les informations requises par le protocole doivent être consignées sur les cahiers d’observation et une explication doit être apportée pour chaque donnée manquante. Les données devront être recueillies au fur et à mesure qu'elles sont obtenues, et transcrites dans ces cahiers de façon nette et lisible.

Les données erronées relevées sur les cahiers d'observation seront clairement barrées et les nouvelles données seront copiées, à côté de l'information barrée, accompagnées des initiales, de la date et éventuellement d’une justification par l’investigateur ou la personne autorisée qui aura fait la correction.

## Saisie et produit des données

Les données patients seront recueillies dans le CRF papier au fur et à mesure de l’étude.

Les données d’imagerie seront anonymisées dans le logiciel OSIRIX / HOROS avec suppression des champs relatifs à l’identité : selon la norme DICOM, les principaux champs sont : PatientName (remplacé par le numéro d’étude), PatientID (remplacé par le numéro d’étude), PatientBirthdate, PatientStudyDate, PatientBirthName et PatientsMother’sBirthName. Les champs PatientAge et PatientSex seront conservés.

Les données anonymisées seront envoyées au centre de recherche de GE medical system par un protocole sécurisé et crypté (secure FTP).

## CNIL

Cette étude entre dans le cadre de la « Méthodologie de Référence » (MR-001) en application des dispositions de l’article 54 alinéa 5 de la loi n°78-17 du 6 janvier 1978 modifiée relative à l’informatique, aux fichiers et aux libertés. Ce changement a été homologué par décision du 5 janvier 2006. Le CHR d’ORLEANS, promoteur de l’étude, a signé un engagement de conformité à cette « Méthodologie de Référence » le 10 mars 2017.

## Archivage

Les documents suivants seront archivés par le nom de l’étude dans les locaux de la direction de la recherche du CHR d’ORLEANS jusqu’à la fin de la période d’utilité pratique.

Ces documents sont :

- Protocole et annexes, amendements éventuels,
- Formulaires d’information
- Données individuelles (copies authentifiées de données brutes)
- Documents de suivi
- Analyses statistiques
- Rapport final de l’étude

A l’issue de la période d’utilité pratique, l’ensemble des documents à archiver sera placé sous la responsabilité du Promoteur pendant 25 ans après la fin de l’étude conformément aux pratiques institutionnelles.

Aucun déplacement ou destruction ne pourra être effectué sans l’accord du Promoteur. Au terme des 25 ans, le promoteur sera consulté pour destruction. Toutes les données, tous les documents et rapports pourront faire l’objet d’audit ou d’inspection.

Comme pour l’examen standard, les données d’imagerie de l’étude seront stockées et archivées dans le PACS de l’établissement.

# Financement et assurance

## Budget de l’étude

Le CHR D’ORLEANS prend en charge les frais liés à la recherche pour ce qui concerne :

- Examens relatifs au protocole : scintigraphie avec le nouveau collimateur
- Temps médical
- Temps statisticien
- Tems chef de projet
- Contrôle qualité par un ARC mandaté par le promoteur
- Assurances

GE medical system fournira les deux collimateurs nécessaires à l’étude.

## Assurance

Le CHR d’ORLEANS a souscrit pour toute la durée de l'étude une assurance auprès de la SHAM garantissant sa propre responsabilité civile ainsi que celle de tout médecin impliqué dans la réalisation de l'étude.

Il assurera également l'indemnisation intégrale des conséquences dommageables à la recherche pour la personne qui s'y prête et ses ayants droit, sauf preuve à sa charge que le dommage n'est pas imputable à sa faute ou à celle de tout intervenant, sans que puisse être opposé le fait d'un tiers ou le retrait volontaire de la personne qui avait initialement consenti à se prêter à la recherche.

# Faisabilité de l'étude

La file active des patients du service de médecine nucléaire se compose d'environ 50 rendez-vous de scintigraphie osseuse par semaine, 15 scintigraphies pulmonaires par semaine, 3 scintigraphies cérébrales au Datscan par semaine, 5 scintigraphies thyroïdiennes et 2 scintigraphies parathyroïdiennes par semaine.

La fiche d'information de participation à l'étude sera envoyée à tous les patients convoqués dans le service. Devant l'absence de risque pour le patient et la faible contrainte liée à l'étude, on peut supposer qu'au moins un patient sur 10, soit 5 patients par semaine seront d'accord pour y participer. L'ensemble des médecins du service (cinq médecins), pourront inclure les patients. Ainsi, l'objectif de 70 patients sur une durée de 12 mois après acceptation de l'étude de semble tout à fait adapté à notre activité.

# Règles relatives à la publication

Les communications et rapports scientifiques correspondant à cette étude seront réalisés sous la responsabilité de l’investigateur principal coordonnateur de l'étude avec l’accord des investigateurs responsables. Les coauteurs du rapport et des publications seront les investigateurs et les cliniciens impliqués, au prorata de leur contribution à l’étude, ainsi que le biostatisticien et les chercheurs associés.

Les règles de publications suivront les recommandations internationales (N Engl J Med, 1997; 336 :309-315).

L’étude sera enregistrée sur un site web en libre accès (Clinical trial) avant l'inclusion du 1^er^ patient dans cette étude.

# Bibliographie

Evaluation of a novel collimator for molecular breast tomosynthesis. Gilland et al. Med Phys 2017 Nov;44(11):5740-5748

Comparison of myocardial perfusion imaging using thallium-201 between a new cadmium-zinc-telluride cardiac camera and a conventional SPECT camera. Songy et al, [Clin Nucl Med.](https://www.ncbi.nlm.nih.gov/pubmed/21825848) 2011 Sep;36(9):776-80

# Liste des annexes

*bordereau de recueil de données / questionnaires*

Marquage CE du nouveau collimateur :


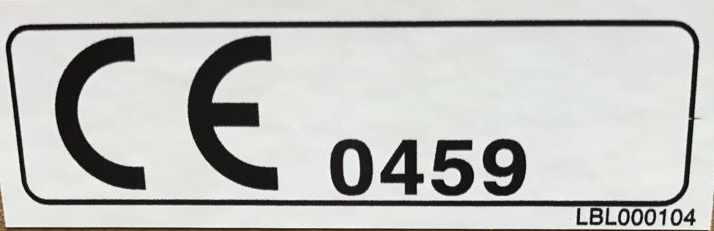

Supplement: S2 File — (DOCX) [file pone.0222490.s002.docx]
