# Supplementary material for: Clinical evaluation of General Electric new Swiftscan solution in bone scintigraphy on NaI-camera: A head to head comparison with Siemens Symbia
Source: PLoS One. 2019 Sep 19;14(9):e0222490. doi: 10.1371/journal.pone.0222490 (PMC6752842; doi:10.1371/journal.pone.0222490)

Clinical study involving the human person and involving only minimal risks and constraints (category 2 of L1121-1)

LEHRS

Comparison of a new LEHRS collimator (General Electric - GE Healthcare) vs Siemens LEHR on image quality in scintigraphy

Version 1.0 - 01/02/2018

PROPONENT		
**Orléans Hospital Center (CHR)**<br>CS 8670945067<br>Orléans Cedex 02		
**PRINCIPAL INVESTIGATOR**		
Dr METRARD Gilles<br>Nuclear medicine department<br>CHR Orléans - 14 , avenue de l'hôpital 45067<br>Orléans cedex 2 - FRANCE		

Table of Contents

Executive Summary 4

1. General informations........ 8

 1.1. Project managers 8

2. Context and scientific justifications 9

 2.1. Pathology description 10

 2.2. Description of the strategy studied 11

 2.3. Summary of results of available non-clinical and clinical trials 13

3. Research objectives 13

 3.1. Main objective 13

 3.2. Secondary objectives 13

 3.3. Main evaluation criteria 13

 3.4. Secondary evaluation criteria 14

4. Study population 14

 4.1. Inclusion criteria 14

 4.2. Criteria for non-inclusion 15

 4.3. Recruitment procedures 15

 4.4. Benefit / risk 15

5. Research design 16

 5.1. Research methodology 16

 5.2. Specific procedures for research 16

 5.3. Study 16

 5.4. Research duration 17

 5.5. Measures taken to reduce and avoid bias 17

 Data collected in the observation bookle 18

 5.6. Description of the rules for permanent or temporary cessation 18

6. Products administered 19

 6.1. Authorized treatments 19

 6.2. Unauthorized treatments 19

 6.3. Emergency treatment 19

7. Security assessment 19

7.1. Procedures for recording and reporting adverse events 19
8. Statistics 20
9. Right of access to data and documents 22
9.1. Access to data ....ii.. 22
9.2. Documents 22
9.3. Confidentiality of data 22
10. Quality control and assurance.. 23
11. Ethical considerations ....
11.1. Committee for the Protection of Persons... 23
11.2. Substantial changes 24
11.3. Patient information and agreement 24
11.4. Registration in the National Register of Persons Suitable for Biomedical Research 24
12.Bibliography.. 27
13 List of appendices 27

AGREEMENT OF THE PROTOCOL

INVESTIGATOR'S SIGNATURE

I have read all the pages of the clinical trial protocol of which ORLEANS CHR is the promoter. I confirm that it contains all informations necessary for the clinical resear ch. I undertake to realize the trial in accordance with the protocol, terms and con ditions defined therein. I undertake to do the research in accordance with:

- the principles of the "Declaration of Helsinki",
- the rules and recommendations of international (ICH) and French good clinical practice (rules of good clinical practice for biomedical research involving medicinal products for human use)
- national legislation and regulations relating to clinical trials,
- compliance with the EU Clinical Trials Directive[2001/20/EC].

I also undertake to ensure that investigators and other qualified members of my team have access to copies of all study documents to work in compliance with the study protocol.

NAME : Dr G. METRARD

Signature: Date :____________________

In accordance with the provisions of the law relating to data processing, files and freedoms, you have the right to access and rectify computer data concerning you at any time (law n° 2004-801 of 6 August 2004 amending law n° 78-17 of 6 January 1978 relating to data processing, files and freedoms).

PROMOTER'S SIGNATURE

Promoter :

NAME: Antoine LEBRERE
Director of Research

Signature : Date :____________________

EXECUTIVE SUMMARY

TITLE	Comparison of a new GE LEHRS vs Siemens LEHR collimator on image quality in scintigraphy.
ACRONYM	LEHRS
PROPONENT	**CHR ORLEANS**<br>14 avenue de l'hôpital<br>45067 Orléans Cedex 02
PRINCIPAL INVESTIGATOR	**Dr Gilles METRARD**<br>**CHR ORLEANS**<br>Nuclear medicine department<br>14 avenue de l'hôpital<br>45067 Orléans Cedex 02
PROTOCOL VERSION	V1.0 of February 1, 2018
RATIONALE / CONTEXT	Performances of a monophotonic scintigraphic examination with a gamma camera requires the use of a collimator. This perforated grid is the subject of industrial studies to find the best compromis e between, on the one hand, the size and number of "holes" (determining sensitivity) and, on the other hand, the thickness and length of septa (conditioning the resolution). These parameters are inversely linked because the increase of septa thickness reduces the aperture surface.
MAIN OBJECTIVE	Compare image quality obtained with the new LEHRS GE collimator versus a standard Siemens LEHR collimator in bone scan studies.
SECONDARY OBJECTIVES	Evaluation of image quality in pulmonary, thyroid, parathyroid and brain scans with DaTSCAN.<br><br>Performances evaluation on physical measurement phantoms.
MAIN JUDGMENT CRITERION	Rate of images obtaining a score greater than or equal to 4 on visual image quality analysis (on a 5-point LIKERT scale).
SECONDARY JUDGMENT CRITERIA	Signal-to-noise ratio measurements on patient acquisitions.<br><br>Physical performances of the new LEHRS collimator using physical measurement phantoms.
METHODOLOGY / STUDY DESIGN	Monocentric, comparative, non-inferiority study with intra-patient comparisons
CRITERIA FOR INCLUSION OF	Patients referred to the department for bone, lung, thyroid,

SUBJECTS	parathyroid or brain scans with DaTSCAN.
CRITERIA FOR NON-INCLUSION OF SUBJECTS	Children, pregnant women or women at risk of pregnancy, Patients under guardianship or under trusteeship, known renal failure, patients with pain, patients who cannot have a standard good quality scintigraphic examination.
STRATEGIES / PROCEDURES	The patient's scans will be performed on 2 separate devices and the images obtained will be compared.
NUMBER OF PATIENTS	85 patients including 70 for bone scans
DURATION OF THE SEARCH	Duration of the inclusion period: 1 year<br><br>Duration of participation for each patient: less than half a day.<br><br>Total duration of the study: 12 months (data inclusion and analysis)
EXPECTED BENEFITS	As individual for the patient: likely to improve the image quality of his examination, interpretation of the examination by 2 expert doctors.<br><br>Collectively: improvement of image quality of scintigraphic exams with this collimator allowing to reduce the duration of exam and/or the injected activity of radiopharmaceutical drugs (improvement of patient dosimetry).

LIST OF ABBREVIATIONS

Table to be completed according to the content of the following protocol

ANSM	National Agency for the Safety of Medicines and Health Products
MA	Marketing Authorization
ARC	Clinical Research Associate
BPC	Good Clinical Practices
CPP	Committee for the Protection of Persons
CNIL	Commission Nationale de l'Informatique et des Libertés
CRF	Case Report Form (observation book)
EvIG	Serious Adverse Event
ISG	Serious Adverse Effect
EIGI	Unexpected Serious Adverse Effect
GE	General Electric Healthcare
ICH	International Conference on Harmonization
FDI	Nurse State graduate
INSERM	National Institute of Health and Medical Research
LEHR	Low Energy High Resolution
MR	Reference Methodology
CPR	Summary of Product Features
SUSAR	Suspected Unexpected Serious Adverse Reaction
TEC	Clinical Study Technician
CT SCAN	Computed tomography

1. GENERAL INFORMATION

1.1. PROJECT MANAGERS

Title of the study: LEHRS<br>Comparison of a new LEHRS collimator (General Electric - GE Healthcare) vs Siemens LEHR on image quality in scintigraphy	
**PROMOTER :**	**CHR Orléans**<br>14 avenue de l'hôpital<br>ORLEANS
**INVESTIGATOR COORDINATOR :**	**Dr Gilles METRARD**<br>Nuclear Medicine Department<br>CHR ORLEANS
**ASSOCIATE INVESTIGATORS :**	**Dr. Matthieu BAILLY**<br>**Dr Hélène BESSE**<br>**Dr. Sabine GAUVAIN**<br>**Dr Sofiane MOUZOUNE**
**METHODOLOGIST / BIOSTATISTICIAN :**	**Dr Antoine VALERY**<br>CHR ORLEANS

2. CONTEXT AND SCIENTIFIC JUSTIFICATIONS

Scintigraphic examinations allows to map, in vivo, the biodistribution of a radiopharmaceutical drug, an analogue of a molecule of interest.

Image of the distribution, in the body, of the radiopharmaceutical drug is obtained by detecting the gamma rays emitted by the radioelement used for its marking.

The addition of a collimator (perforated grid) in front of the detectors is needed to select the most informative gamma photons whose direction is orthogonal to the detector.

The choice of the collimator used is based on a compromise between two parameters:

- The thickness and depth of septa. Higher is the energy of the emitted gamma photon (specific to each radioelement), higher is the probability that this photon will pass through material, especially the walls (septa) of the collimator. This septal penetration reduces the spatial resolution and image quality. For this reason, collimators are classified into three main categories, according to the increasing thickness of their septa : low energy (LE), mean energy (ME) and high energy ("high energy" HE)

- The width and number of holes. Large holes increase the sensitivity of the gamma camera but r educe the number or thickness of septa for a constant detector surface. A good sensitivity allows to reduce the examination time but with an alteration of the resolution.

The majority of nuclear medicine examinations use radiopharmaceutical drugs (labelled with technetium 99m, emitting gamma photons, 140keV energy peak) and require the use of low energy collimators (LE). There are large versions of this kind of collimators, the first one promotes sensitivity and the second one promotes resolution (LEHR for "Low Energy High Resolution").
We want to evaluate a new intermediate kind of collimators (LEHRS for "Low Energy High Resolution and Sensitivity", GE) wich is design to enhance sensitivity without compromising resolution. The expected degradation of the resolution is compensated by computer correction algorithms.

2.1. PATHOLOGY DESCRIPTION

Bone scan is a common practice examination in nuclear medicine. The radiopharmaceutical drug used in this study is a technetium-99m labelled biphosphonate (in our department, it is hydroxydiphosphonate or HDP-99mTc).

It is an examination that significantly quantifies bone anabolism in response to pathologies and allows a whole body scan in a single examination.

Bone diseases are multiple and can be of tumor, traumatic, rheumatic or infectious origin.
Indications for bone scans are varied: rheumatological and orthopaedic (exploration of diffuse bone pain, assessment of inflammatory rheumatism, dating of fractures, research of algoneurodystrophy, exploration of pain on prostheses, in particular research on loosening...), oncological (assessment of extension of bone tumours, in particular tumours with osteocondensing metastases as in prostate cancers, assessment of extension of sarcoma and other bone tumours...)

The main advantages of bone scanning are based on good accessibility of the technique (unlike MRI), its exploration of the whole body, in a reduced examination time (whole body scanning in about 15 minutes), for a relatively low irradiation compared to the diagnostic contribution of the technique. For an adult of 70 kg, the effective dose is 4mSv; for comparison, the natural irradiation in Orléans (related to cosmic and terrestrial radiation from the soil) is about 1 to 2 mSv per year. A thoraco-abdomino-pelvic scanner delivers an average effective dose of 18 mSv according to the 2010 IRSN report "Doses delivered to patients in CT and conventional radiolo gy".
It is also important to note that, unlike radiology examinations, irradiation of scintigraphy is related to radiopharmaceutical drug posology and therefore does not increase with the number of scintigraphic acquisitions performed.

2.2. DESCRIPTION OF THE STRATEGY STUDIED

Exam protocol will be conforme to French and international medical societies recommandations. According to indication, early images will be realized after radiopharmaceutical drug injection. Late bone scan images will systematically be realized 2 hours after injection (for the radiopharmaceutical drug used in our department), mentioned as whole-body scan. This acquisition is often complemented by additional planar acquisitions, focused on a region of interest (most often the painful area and/or pathologic bone uptake).

In some cases, a tomoscintigraphy will be performed, allowing to obtain 3D volume scintigraphy images, improving the sensitivity of detection of small abnormal bone fixations. This acquisition of tomoscintigraphy will be associated with CT scans for more accurate anatomical localization.

In addition to this standard examination, the patient's participation in the research protocol will involve the creation of complementary images with the new collimator : a second whole body

scan, a static acquisition, and in cases where it has been requested for the standard examination, a tomoscintigraphy (without CT in this case, to maintain dosimetry equivalent to the standard examination).

2.3. SUMMARY OF RESULTS OF AVAILABLE NON-CLINICAL AND CLINICAL TRIALS

No study has yet been realized to evaluate this new collimator.

Few data from the literature are available on the evaluation of new collimators in nuclear medicine. Most studies are medical physics studies with evaluation of performances in terms of sensitivity and resolution on physical measurement objects (e.g., evaluation of a new collimator in mammary tomosynthesis, Gilland et al. Med Phys 2017 Nov;44(11):5740-5748). An evaluation of the old and new collimator on physical measurement objects is planned in our study.

Our research work can be compared to the studies realized after the commercialization of solid-state nuclear medicine cameras (known as CZT, for cadmium-zinc-telluride). For example, in the retrospective study conducted by the CCN team (Songy et al, Clin Nucl Med. 2011 Sep;36(9): 776-80), 153 patients received their cardiac scans on an older generation camera and then on the new CZT camera. The data were analyzed retrospectively, comparing image quality and also the final diagnosis.

3. RESEARCH OBJECTIVES

3.1. MAIN OBJECTIVE

Compare image quality obtained with the new LEHRS GE collimator with that obtained with a standard Siemens LEHR collimator on bone scan examinations.

3.2. SECONDARY OBJECTIVES

Performance evaluation on physical measurement phantoms.

Evaluation of image quality in pulmonary, thyroid, parathyroid and brain scans with DaTSCAN.

3.3. MAIN EVALUATION CRITERIA

The main evaluation criterion will be the image quality evaluation on bone scanning obtained with the new LEHRS GE collimator vs. standard Siemens LEHR collimator (two expert nuclear physicians).

There is no standard criteria for evaluating image quality in scintigraphy. A good quality scintigraphic image (sensitive and good spatial resolution) will present good bone fixation, with low background noise, well defined bone surfaces, and good visualization of interosseous spaces.

We will therefore use a 5-point Likert scale :

5 Diagnostic quality - Excellent image quality and resolution (excellent bone fixation, almost non-existent background noise, good visualization of bone surfaces and interosseous spaces)

4 Diagnostic quality - Good image quality and resolution (good bone fixation, low background noise, good visualization of bone surfaces and interosseous spaces)

3 Diagnostic quality - Acceptable image quality and resolution (good bone fixation, moderate background noise, blurred visualization of bone surfaces and interosseous spaces)

2 Suboptimal quality - Limited clinical information (poor bone fixation, moderate to high background noise, poor visualization of bone surfaces and interosseous spaces)

1 Non-diagnostic quality (poor bone fixation or almost no bone fixation, high background noise)

For each patient, this score will be established for each image type and for each collimator, e.g. whole body scanning, static imaging, tomoscintigraphy (if performed).

The percentage of examinations judged to be of good quality (scores 4 and 5) will be compared for each collimator and each image type.

3.4. SECONDARY EVALUATION CRITERIA

The secondary evaluation criteria will be:

- Physical measurements of collimator performances on phantoms.
- Evaluation of the signal-to-noise ratio in bone scan images (sacroiliac fixation ratios / soft tissue on whole body scanning) obtained using the two sets of collimators.
- A quantified assessment (Likert scale) of the image quality obtained in lung, thyroid, parathyroid or brain scintigraphic examinations at DaTSCAN.
- An evaluation of the signal-to-noise ratio in brain scan images at DaTSCAN, by comparing the striata/occiput fixation ratios obtained using the two sets of collimators, after automatic analysis and segmentation in DaTQUANT (GE) software.

4. STUDY POPULATION

4.1. INCLUSION CRITERIA

Any patient referred for bone, lung, thyroid, parathyroid or brain scans with DaTSCAN.

4.2. CRITERIA FOR NON-INCLUSION

- Minors,
- Pregnant women or women at risk of pregnancy (beta-HCG dosage will be performed 48 hours before examination for patients in the second part of the cycle or in the absence of contraception),
- Patients with known renal insufficiency,
- Patients with pain, EVA >4,
- Patients who cannot benefit from a standard good quality scintigraphic examination (agitated patients...)
- Person under guardianship or curatorship

4.3. RECRUITMENT PROCEDURES

Study will be proposed to all eligible patients, referred for bone, lung, thyroid, parathyroid or brain scans with DaTSCAN. Each patient will receive the study information leaflet.

The patient will be seen by a nuclear medical physician of the team as soon as he arrives in the nuclear medicine department, before exam. The study protocol and the various informations will then be explained to him.

Informational documents will be left for the patient to continue to review, in greater detail, during the waiting time before acquisitions are completed (approximately 2 hours after the injection of the radiopharmaceutical drug).

If at the end of this additional reflection period, the patient still agrees to participate to the study, his consent form will be collected by the manipulator, who will put it in the study folder. In case of non agreement, only standard examination will be performed and the patient will not participate to the study.

4.4. BENEFIT / RISK

Participation to this study does not create any additional risk for the patient, since the completion of the ^{2nd} scintigraphy does not change the posology, mode and frequency (single injection) of the injected radiopharmaceutical drug.

The patient will maybe benefit from image quality improvement and an exam review by 2 expert physicians. The only constraint for the patient will be an extended examination time of 15 to 30 minutes, compared to a "standard" examination, without any further invasive procedures or additional irradiation.

Collectively, image quality improvement with this collimator could reduce the duration of exams and/or the posology of radiopharmaceutical drugs (improvement of patient dosimetry).

5. Research design

5.1. Research methodology

Monocentric, comparative, non-inferiority study, with intra-patient comparisons (acquisition vs LEHR Siemens reference collimator, for each patient).

Interpretation of image quality in double-blind mode.

5.2. Specific procedures for research

The management of patients included in this study is based on the standard conventional management.

It will simply be carried out, with the radiopharmaceutical drug already injected for the standard examination, a larger number of acquisitions (taken with the set of two collimators to be evaluated), but without additional irradiation.

In the case of CT scan performed for the standard examination (depending on the clinical indication of the examination), only one CT scan will be performed for the two acquisitions.

5.3. Study

5.3.1. Inclusion

The inclusion and non-inclusion criteria will be checked during a medical consultation with the patient. This consultation will be carried out as soon as the patient arrives in the department or, at the latest, before the completion of late acquisitions (approximately 2 hours after the injection).

Information and consent will be obtained during this consultation with one of the doctors on duty. During this consultation, the various criteria for inclusion and non-inclusion will also be checked.

After a decision period, if patient participate in this study, the various documents will be collected in the patient's file, at the latest before the late pictures of the standard scintigraphy examination are taken.

At any time, the patient may, if patient indicates his refusal, leave the study. In this case, only acquisitions necessary for the standard examination will be made.

5.3.2. Patient follow-up

No patient follow-up, other than that required for the pathology explored in standard scintigraphy, will be required for the study.

5.4. RESEARCH DURATION

The inclusion period will take place over a period of 12 months after the CPP has given its approval for the study to be carried out and the equipment to be made available (collimator...).

The total duration of the patient's participation will not exceed half a day.

5.5. MEASURES TAKEN TO REDUCE AND AVOID BIAS

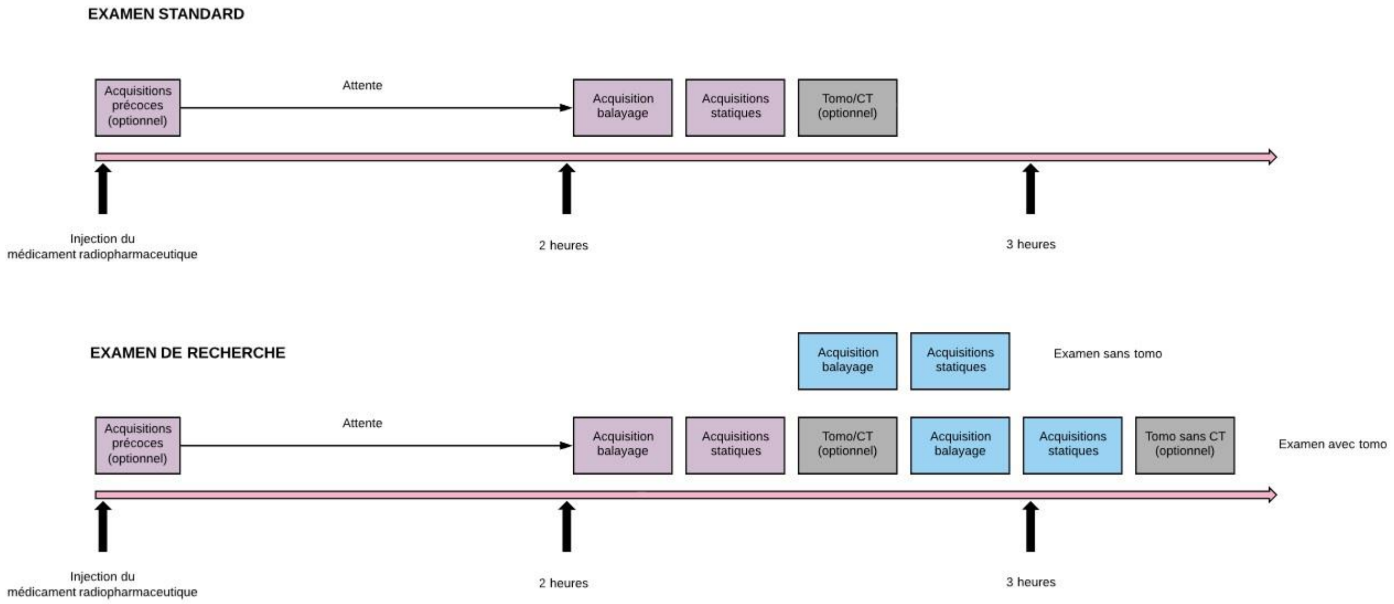

5.5.1. Drawing of lots

A draw will not be carried out because the standard examination will always be carried out first in order not to cause a loss of chance if the examination is stopped (patient with pain...). This also allows the patient to refuse to participate in the study at the end of his or her standard examination. The protocol will be adapted to compensate for the delay between the two examinations (increased acquisition time in proportion to the radioactive decrease).

Patients will be included consecutively. Images will be realized first on the GE camera, equipped with the LEHRS collimator, or on the Siemens camera, with LEHR collimator, depending on his initial appointment slot. However, when 50% of the study population has been achieved on one of the two cameras, the inclusions will only continue on the other.

5.5.2. Blinding method

The imaging data will be anonymized, by a person who will not be part of the image reviewers, to remove all information related to the patient's identity from the DICOM fields of the image (metadata). A number will be assigned to each patient, using a randomization table. The patient's identity - anonymization number correspondence will be collected in a specific database.
Images will be read at the end of the inclusions, from the anonymized data, by two senior doctors, on a post-processing console. The anonymization method will not identify the patient's identity or the type of device used (LEHR or LEHRS collimator).

5.6. DATA COLLECTED IN THE OBSERVATION BOOKLET

- Patient data: age, sex, weight, BMI.
- Radiopharmaceutical drug-related data: radiopharmaceutical drug name, injected activity, time and injection site.
- Data related to acquisitions: acquisition times, physical parameters (peak energy, fenestration, acquisition time, time per projection and number of projections if tomoscintigraphy...)

As with the standard review, the study imaging data will be stored and archived in the institution's PACS.

5.7. DESCRIPTION OF THE RULES FOR PERMANENT OR TEMPORARY CESSATION

5.7.1. Stopping a person's participation in research

Subjects may withdraw their consent and ask to leave the study at any time and for any reason. In the event of early discharge, the investigator should document the reasons as fully as possible.

The investigator may temporarily or permanently suspend the participation of a subject in the study for any reason that would best serve the interests of the subject, particularly in the event of painful symptomatology or major claustrophobia during acquisitions.

A patient's discharge from a study will not change his or her usual management of the disease.

5.7.2. Stop some or all of the research

The study may be stopped prematurely if unexpected, serious adverse events occur that require a review of the strategy profile. Similarly, unexpected events or new product information, in the light of which the objectives of the study or clinical program are unlikely to be achieved, may lead the sponsor to prematurely discontinue the study.

The ORLEANS CHR reserves the right to discontinue the study at any time if it appears that the inclusion objectives have not been met.

6. PRODUCTS ADMINISTERED TO RESEARCH SUBJECTS

An injection of radiopharmaceutical drug, necessary for the standard scintigraphic examination, will be carried out, as in current practice. There will be no additional injection related to the research protocol. The summaries of product characteristics (RCP) of the radiopharmaceuticals used in this study are presented in the Appendix.

6.1. AUTHORIZED TREATMENTS

Usual treatment of the patient, analgesics....

6.2. UNAUTHORIZED PROCESSING

No processing is involved. Any treatment not authorized for the standard examination will result in its cancellation and, in fact, participation in the study (verification of the latest chemotherapy dates, injection of biphosphonates, synthetic antithyroid agents and thyroid hormones...)

6.3. EMERGENCY TREATMENT

Not concerned. No major adverse reactions are documented for radiopharmaceuticals used in the standard review.

7. SECURITY ASSESSMENT

7.1. PROCEDURES IN PLACE FOR RECORDING AND REPORTING ADVERSE EVENTS

As this is a category 2 study, no particular vigilance related to the research protocol will required.

However, the monitoring and reporting of incidents and incident risks resulting from participation in the study will be reported through usual channels. As the collimator and the software used are CE marked, any adverse events will be the subject of a material report by ORLEANS CHR.

7.2. Supervisory Committee

Since participation in the study did not pose any risk to the patient, it was not considered necessary to set up a monitoring committee.

7.3. Methods and duration of follow-up of individuals following the occurrence of adverse eventss

Each adverse event will be monitored until it is fully resolved (stabilized to a level acceptable to the investigator or returned to its previous state) even if the patient has been discharged from the trial.

8. Statistics

For statistical analysis, data were collected on Excel spread sheet and analyzed using the Statistical Package for Social Sciences (SPSS; IBM SPSS, Armonk, NY, USA).
Sample size was calculated with expected performances on phantom.
For hypothesis testing, a non-parametric Wilcoxon signed rank test was used for categorical and non-Gaussian distribution variables (variable's distribution was tested with a Kolmogorov-Smirnov normality test).
A non-inferiority test was also performed.
Inter-observer agreement was assessed with a Cohen's kappa test. The level of agreement was classified into six Landis and Koch categories

9. RIGHT OF ACCESS TO SOURCE DATA AND DOCUMENTS

9.1. ACCESS TO DATA

In accordance with GCP:

- the sponsor is responsible for obtaining the agreement of all parties involved in the research to ensure direct access to all research sites, source data, source documents and reports for the purpose of quality control and audit by the sponsor,

- the investigators shall make available to persons responsible for monitoring, quality control or audit of biomedical research, the individual documents and data strictly necessary for such control, in accordance with the laws and regulations in force (Articles L. 1121-3 and R. 5121-13 of the Public Health Code).

9.2. SOURCE DOCUMENTS

Source documents being defined as any original document or object used to prove the existence or accuracy of a data or fact recorded during the clinical study will be kept for 25 years by the investigator or by the hospital in the case of a hospital medical file.

9.3. CONFIDENTIALITY OF DATA

In accordance with the provisions concerning the confidentiality of data to which persons responsible for quality control of biomedical research have access (Article L. 1121-3 of the Public Health Code), in accordance with the provisions relating to the confidentiality of information concerning in particular the nature of the products, the tests, the persons who lend themselves to them and the results obtained (Article R. 5121-13 of the Public Health Code), persons having direct access shall take all necessary precautions to ensure the confidentiality of information relating to the products, the tests,

the persons who lend themselves to them and in particular with regard to their identity and the results obtained.

These persons, like the investigators themselves, are subject to professional secrecy (under the conditions defined by Articles 226-13 and 226-14 of the Criminal Code).

During or at the end of biomedical research, data collected on suitable individuals and transmitted to the sponsor by investigators (or any other specialized stakeholders) will be made anonymous.

Under no circumstances should they disclose in plain language the names of the persons concerned or their addresses.

Only the first letter of the subject's name and the first letter of the subject's first name will be registered, accompanied by a study-specific coded number indicating the order in which the subjects are included. Patient images will be anonymized, using a specific feature of the image processing console.

The sponsor shall ensure that each person who is suitable for the research has given his or her written consent to access the individual data concerning him or her that are strictly necessary for the quality control of the research.

10. QUALITY CONTROL AND ASSURANCE

A Clinical Research Associate (CRA) mandated by the sponsor will ensure the successful completion of the study.

The investigator and members of his team agree to make themselves available during Quality Control visits carried out at regular intervals by the Clinical Research Associate. During these visits, the following elements will be reviewed:

- express agreement
- compliance with the study protocol and procedures defined therein

On the other hand, the investigators undertake to accept the quality assurance audits carried out by the promoter as well as the inspections carried out by the Competent Authorities. All data, documents and reports are subject to regulatory audits and inspections without the possibility of medical confidentiality.

11. ETHICAL CONSIDERATIONS

11.1. COMMITTEE FOR THE PROTECTION OF PERSONS

The protocol and the study information form will be submitted for opinion to a People's Protection Committee designated by drawing lots.

The notification of the CPP's favourable opinion will be forwarded to the study sponsor and the Competent Authority.

11.2. Substantial changes

If the investigator makes a substantial change to the protocol, it will be approved by the sponsor. The latter must obtain a favourable opinion from the PPC before implementing it. A new consent from the research participants will be obtained if necessary.

11.3. Patient information and express consent

Patients will be informed in a complete and fair manner, in understandable terms, of the objectives and constraints of the study, the possible risks involved, the necessary surveillance and safety measures, their rights to refuse to participate in the study or the possibility of withdrawing at any time.

All this information is provided on an information form given to the patient. The express and written consent of the patient will be obtained by the investigator, or a physician representing the patient, before final inclusion in the study.

11.4. Registration in the National Register of Persons Suitable for Biomedical Research

This registration is not part of the research

12. BIBLIOGRAPHY

Evaluation of a novel collimator for molecular breast tomosynthesis. Gilland et al. Med Phys 2017 Nov444(11):5740-5748

Comparison of myocardial perfusion imaging using thallium-201 between a new cadmium-zinc-telluride cardiac camera and a conventional SPECT camera. Songy et al, Clin Nucl Med. 2011 Sep436(9):776-80

13. LIST OF APPENDICES

data collection form / questionnaires

CE marking of the new collimator:

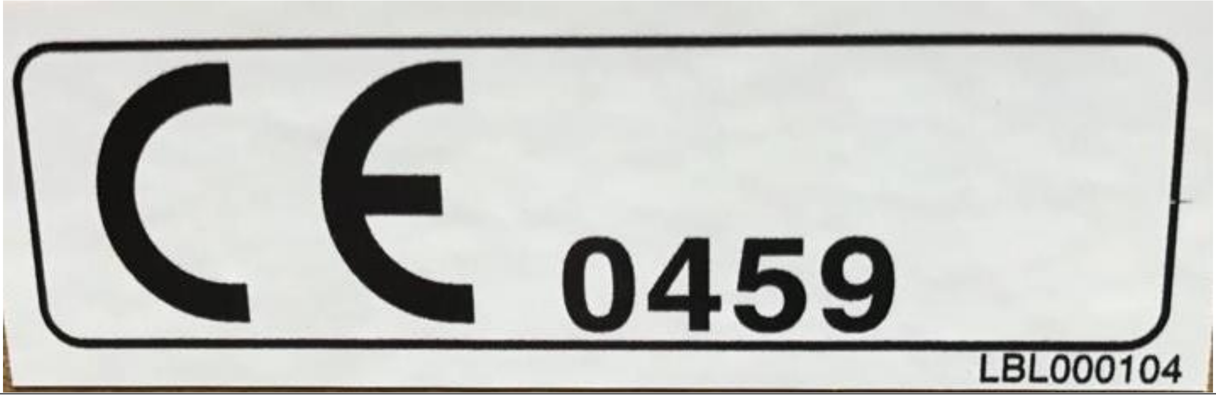

Supplement: S3 File — (PDF) [file pone.0222490.s003.pdf]
