# Supplementary material for: Clinical evaluation of General Electric new Swiftscan solution in bone scintigraphy on NaI-camera: A head to head comparison with Siemens Symbia
Source: PLoS One. 2019 Sep 19;14(9):e0222490. doi: 10.1371/journal.pone.0222490 (PMC6752842; doi:10.1371/journal.pone.0222490)
Supplement: S4 File — (DOCX) [file pone.0222490.s004.docx]

| **[Brief Title](https://register.clinicaltrials.gov/prs/html/definitions.html#BriefTitle)** | LEHRS |
| --- | --- |
| [**Official Title**](https://register.clinicaltrials.gov/prs/html/definitions.html#OfficialTitle) | Comparison of image quality between new collimator LEHRS (General Electric – GE Healthcare) vs Siemens LEHR on bone scintigraphy. |
| [**Brief Summary**](https://register.clinicaltrials.gov/prs/html/definitions.html#BriefSummary) | Comparison of quality image with a new LEHRS GE collimator vs standard LEHR Siemens collimator on bone scintigraphy. |
| [**Detailed Description**](https://register.clinicaltrials.gov/prs/html/definitions.html#DetailedDescription) | Comparison of quality image with a new LEHRS GE collimator vs standard LEHR Siemens collimator on bone scan.  Main objective : Comparison of quality image on bone scintigraphy  Secondary objectives : Quality image on others scintigraphy (lung, DaTSCAN …). |
| **Condition (MESH)** |  |
| **Keywords** | Collimator, bone scintigraphy, image quality, scintigraphy |
| **Arms and interventions** | N/A |
| **Primary purpose** | Treatment  Prevention  Diagnostic  Supportive care  Screening  Health service research  Basic science  Device feasibility  Other |
| [**Study Phase**](https://register.clinicaltrials.gov/prs/html/definitions.html#StudyPhase) | Phase 0  Phase 1  Phase 1/ Phase 2  Phase 2  Phase 2/Phase 3  Phase 3  Phase 4  N/A |
| [**Intervention Model**](https://register.clinicaltrials.gov/prs/html/definitions.html#IntDesign) | Single Group  Parallel  Crossover  Factorial  Sequential |
| [**Number of Arms**](https://register.clinicaltrials.gov/prs/html/definitions.html#NumberOfArms) | N/A |
| **Primary Outcome Measure** | |
| [**Outcome Measure:**](https://register.clinicaltrials.gov/prs/html/definitions.html#Outcomes) | 5 points likert scale to assess visual quality of bone exams. |
| [**Time Frame:**](https://register.clinicaltrials.gov/prs/html/definitions.html#Outcomes) *Le temps doit être mesurable* |  |
| [**Description:**](https://register.clinicaltrials.gov/prs/html/definitions.html#Outcomes) |  |
| **Secondary Outcome Measure** | |
| [**Outcome Measure:**](https://register.clinicaltrials.gov/prs/html/definitions.html#Outcomes) | Quantitative signal-noise-ratios  and quantitative datas on phantom |
| [**Time Frame:**](https://register.clinicaltrials.gov/prs/html/definitions.html#Outcomes) *Le temps doit être mesurable* |  |
| [**Description:**](https://register.clinicaltrials.gov/prs/html/definitions.html#Outcomes) |  |
|  | |
| **Inclusion Criteria** | Refered patients for scintigraphy |
| **Exclusion Criteria** | Under 18 y. old patients  Pregnancy or supposed to be pregnant patients  Kidney failure  Painful patients  Patients under guardianship  Patients in whom a standard exam is not feasible |
| **References**  ***Préciser uniquement le PubMed ID et préciser oui/non si results reference (pour chaque référence)*** |  |
